# Multiplex PCR-based identification of two sympatric stem borer species, *Sesamia cretica* and *Sesamia nonagrioides* (Lepidoptera: Noctuidae)

**DOI:** 10.1093/jisesa/ieaf105

**Published:** 2025-12-19

**Authors:** Fatemeh Karimi, Asadollah Hosseini Chegeni, Zahra Mirzaeipour

**Affiliations:** Department of Plant Protection, Faculty of Agriculture, Lorestan University, Khorramabad, Iran; Department of Plant Protection, Faculty of Agriculture, Lorestan University, Khorramabad, Iran; Department of Plant Protection, Faculty of Agriculture, Lorestan University, Khorramabad, Iran

**Keywords:** Molecular approach, Species diagnosis, *COI* gene, *Sesamia* spp, Iran

## Abstract

Stem borers of the genus *Sesamia* are major pests of cereals in tropical regions. In Iran, *Sesamia cretica* and *Sesamia nonagrioides* significantly affect maize and sugarcane, but distinguishing them at the larval stage is difficult due to morphological similarity. In this study, we developed and optimized a rapid, cost-effective multiplex PCR method for simultaneous identification of both species. Primers designed from GenBank sequences yielded specific bands of 367 bp for *S. cretica* and 438 bp for *S. nonagrioides*. PCR and sequencing confirmed the specificity of the designed primers, while morphological comparison of larvae and reared adults validated the molecular results. This molecular tool supports integrated pest management by improving identification accuracy, which could help reduce unnecessary pesticide applications.

## Introduction

Stem borer moths, especially *Sesamia* species (Lepidoptera: Noctuidae), are among the most destructive pests of tropical and subtropical crops (Pedigo et al. 2021). These pests cause significant damage to cereals, especially in Mediterranean countries, Africa, the Middle East, and South Asia (Li et al. 2024). In Iran, four *Sesamia* species—*Sesamia nonagrioides* (Lefèbvre), *Sesamia cretica* Lederer, *Sesamia rungsi* Boursin, and *Sesamia inferens* (Walker)—are distributed across various regions, mainly southern provinces (Rajaei et al. 2023). *S. cretica* is a major pest of maize and sugarcane, often causing considerable yield losses. *S. nonagrioides* is a well-known pest of maize in southern Europe and northern Africa and is also reported in Iranian maize and sugarcane fields (Ranjbar-e-Aghdam and Kamali 2002, Askarianzadeh et al. 2008).

The larvae penetrate plant stems and damage vascular tissues, which reduces yield and compromises crop quality, and in severe cases can cause complete crop loss (Al-Kahtani et al. 2023, Ebrahimifar et al. 2023). Accurate identification of *S. cretica* and *S. nonagrioides*, at the larval stage, is difficult due to morphological similarities, often resulting in misidentification and suboptimal pest management outcomes (Hévin et al. 2024).

Molecular techniques such as PCR—and particularly multiplex PCR (M-PCR)—are powerful tools for accurate identification at early developmental stages, allowing differentiation between closely related species targeting species-specific genetic markers (Jangra et al. 2020, Andrews et al. 2022, Zhou et al. 2023). Developing such methods for *S. cretica* and *S. nonagrioides* not only enhance pest management strategies but may also help minimize crop losses and enhance productivity. In areas where both species coexist—such as some southern provinces of Iran—accurate identification is critical for implementing targeted and effective pest management programs.

Despite the economic importance of both *S. cretica* and *S. nonagrioides*, no time-saving and low-cost tools have been available for larval-stage identification without rearing or expert morphological analysis. Traditional methods are often inadequate, whereas combining haplotypic analysis with species-specific primers enhances diagnostic reliability in diverse populations (Nagoshi et al. 2006).

While previous studies (Margaritopoulos et al. 2007, Mehravar et al. 2017) have used various molecular markers to identify species of the genus *Sesamia*, primers specifically designed based on haplotype and phylogenetic differences for *S. cretica* and *S. nonagrioides* have not been reported so far. This study presents, for the first time, a newly designed primer set with high discriminatory power, enabling the development of a M-PCR method that can rapidly and simultaneously distinguish between these two species at the larval stage.

## Materials and Methods

### Sample Collection and Preservation

Larval specimens of *S. cretica* and *S. nonagrioides* were collected from maize and sugarcane fields across several localities in Khuzestan Province, southwestern Iran, during the 2025 growing season (early spring to mid-summer 2025). Sampling was done during the growing season and when infestation was observed. Preliminary species identification was based on diagnostic head capsule coloration (lighter in *S. cretica*, darker in *S. nonagrioides*), and in a subset of cases, rearing to adults confirmed identification through forewing coloration and genitalia examination.

### DNA Extraction

To avoid contamination from gut contents, the larval head capsule was used for DNA extraction. DNA extraction was performed using the cetyltrimethylammonium bromide (CTAB) method. Briefly, extraction involved incubation with CTAB buffer, chloroform–isoamyl alcohol separation, precipitation with cold isopropanol, washing with 70% ethanol, and resuspension in nuclease-free water buffer. DNA purity and concentration were evaluated using a NanoDrop spectrophotometer (Thermo Fisher Scientific, United States) and quality was confirmed by 1% agarose gel electrophoresis. Samples with A260/A280 ratios between 1.8 and 2.0 were selected for analysis.

### Primer Design and M-PCR Assay

A total of 73 *COI* sequences (30 *S. cretica* and 43 *S. nonagrioides*) from Iran, North Africa, and southern Europe were downloaded from GenBank for primer design. Multiple sequence alignment using SeaView v5.0.4 (Gouy et al. 2010) identified conserved and variable regions distinguishing two species. The primer-binding sites and alignment overview are shown in Supplementary Fig. S1. Initial primer candidates were designed using GeneDoc software (Nicholas et al. 1997) and finalized using the IDT OligoAnalyzer tool (Integrated DNA Technologies, United States), considering GC content, melting temperature (Tm: ∼47 to 50 °C), and secondary structures. Primer specificity was verified in silico using Primer-BLAST (Ye et al. 2012) to ensure exclusive binding to the target species. For the M-PCR reaction, forward primers specific to each species and a common reverse primer were utilized (Table 1). Primer specificity was assessed using NCBI Primer-BLAST. The designed primers showed perfect matches only with *S. cretica* and *S. nonagrioides* sequences and no alignment with other *Sesamia* species, including *S. inferens*, *S. calamistis*, and *S. albicolor*. BLAST results for nine *S. inferens* sequences (GU681997, KP658205, KT250763, MG838445, MT124086-7, MT734546, MT734554, MT734563, and MT775880) indicated partial mismatches and no predicted amplification, confirming the assay’s high specificity.

**Table 1.** Sequences and properties of species-specific primers designed for the multiplex PCR assay targeting *Sesamia cretica* and *Sesamia nonagrioides*

Primer name	Sequence (5′→3′)	Length (bp)	Melting temp. (°C)	GC content (%)	Target species	Product size (bp)
SC-F	GGT ATA TCC CCC CCT C	16	50.2	62.2	*Sesamia cretica*	367
SN-F	CTA TTA CCA CCA TCC TTA ACC C	22	52.5	45.5	*Sesamia nonagrioides*	438
SR-R	GGT AAA ATT AAA ATA TAA ACT TCT GG	26	47.2	23.1	*Sesamia cretica/Sesamia nonagrioides*	—

SC-F, *Sesamia cretica* forward primer; SN-F, *Sesamia nonagrioides* forward primer; SR-R, shared reverse primer for both species.

### PCR Protocol and Optimization

Initial singleplex PCR assays were performed to test individual primers. Reactions were carried out in a 25 µl volume containing 12.5 µl Taq Master Mix (Amplicon, Denmark), 0.5 µM of each primer, 1 µl of genomic DNA (∼20 ng/µl), and nuclease-free water. The thermal cycling program included an initial denaturation at 94 °C for 4 min, followed by 35 cycles of denaturation (94 °C, 30 s), annealing (50 °C, 45 s), and extension (72 °C, 30 sec.), with a final extension at 72 °C for 10 min. After confirming product specificity, the M-PCR was optimized by combining both species-specific forward primers with the shared reverse primer.

### Gel Electrophoresis

PCR products were separated by electrophoresis on 1.5% agarose gels in 1× TAE buffer and stained with Safe-Red (Intron Biotechnology, Korea). A 100 bp DNA ladder (SinaClon, Iran) was used as a size standard. Gels were visualized under UV illumination using a GelDoc XR+ imaging system (Bio-Rad, United States), and band sizes were analyzed using GelDoc software. All reactions were performed in at least 2 technical replicates and repeated across independent biological samples.

### Sequencing and Morphological Validation

Selected PCR products were purified and sequenced unidirectionally by Codon Company (Iran). Chromatograms were examined and manually edited for base-calling quality using FinchTV v1.4.0 (Geospiza Inc., Seattle, WA, United States). Resulting sequences were compared to reference data using BLASTn (NCBI), and identities were confirmed with ≥99% similarity. Morphological validation was conducted using established taxonomic keys such as Hévin et al. (2024). In several cases, larvae were reared to adulthood under laboratory conditions to confirm diagnostic characters such as wing coloration and male and female genitalia. To verify species identity, Sanger sequencing was performed on 1 representative individual per species, whereas all specimens (*n* = 50) were identified morphologically (larval head capsule coloration and genitalia) prior to molecular analyses.

### Phylogenetic and Haplotype Analyses

All validated sequences from this study (one *S. nonagrioides*, accession number PV646648; one *S. cretica*, accession number PV646697) were submitted to GenBank. Alignments were performed using SeaView, and phylogenetic trees were constructed in MEGA 12 (Kumar et al. 2024) using the Neighbor-Joining and Maximum Likelihood methods with 1,000 bootstrap replicates and in BEAST v2.6 using Bayesian inference. No outgroup was included due to the short sequence length (388 bp), and trees are presented unrooted. Genetic distances were calculated using the Maximum Composite Likelihood model. Intraspecific genetic diversity was assessed using DnaSP v6.12 (Rozas et al. 2017), and haplotype networks were generated using the TCS method in PopART v1.7 (Leigh et al. 2015).

## Results

### Insect Collection and DNA Extraction

A total of 50 larval samples of *S. cretica* and *S. nonagrioides* were collected from maize and sugarcane fields in Khuzestan, Iran. DNA was extracted from head capsules, and quality was confirmed with an A260/A280 ratio of 1.8 to 2.0, as well as by electrophoresis on a 1% agarose gel.

### M-PCR and Gel Electrophoresis

**Fig. 1.**
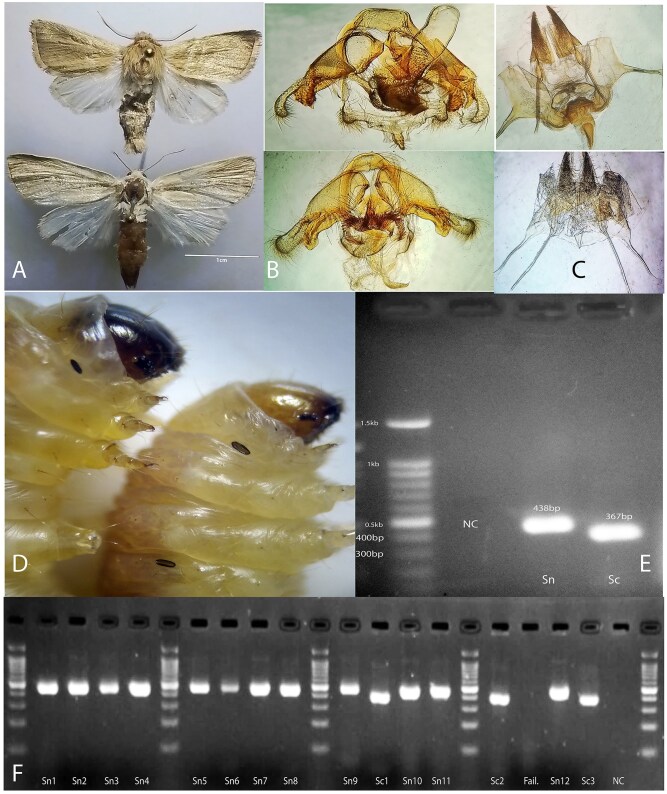
PCR-based identification and morphological comparison of Sesamia species. (A) Adults: S. cretica (below; lighter scales) vs. S. nonagrioides (above; darker scales). (B) Adults’ male genitalia: S. cretica (below) vs. S. nonagrioides (above). (C) Adults’ female genitalia: S. cretica (below) vs. S. nonagrioides (above). (D) Larval head capsules: darker pigmentation in S. nonagrioides (left) vs. lighter in S. cretica (right). (E) Agarose gel showing 438 bp band for S. nonagrioides (Sn) and 367 bp band for S. cretica (Sc). M = 100 bp ladder (key sizes: 300, 400, 0.5kb), NC = negative control. (F) Gel of field samples: Sn1–Sn11 = S. nonagrioides, Sc1–Sc3 = S. cretica, Fail = no amplification, NC = negative control.

M-PCR was used to amplify species-specific regions of the *COI* gene. The designed primers successfully produced diagnostic amplicons of 367 bp for *S. cretica* and 438 bp for *S. nonagrioides* (Fig. 1A). These bands were consistent across both field-collected and laboratory-reared samples (Fig. 1B to D). The alignment of *COI* sequences (Supplementary Fig. S1) confirmed the presence of diagnostic substitutions between the 2 target species, allowing specific primer design. The negative control (no DNA template) yielded no amplification, confirming the specificity of the primers. The results were reproducible, indicating the reliability and accuracy of the PCR assay.

### Phylogeny and Haplotype Structure

**Fig. 2.**
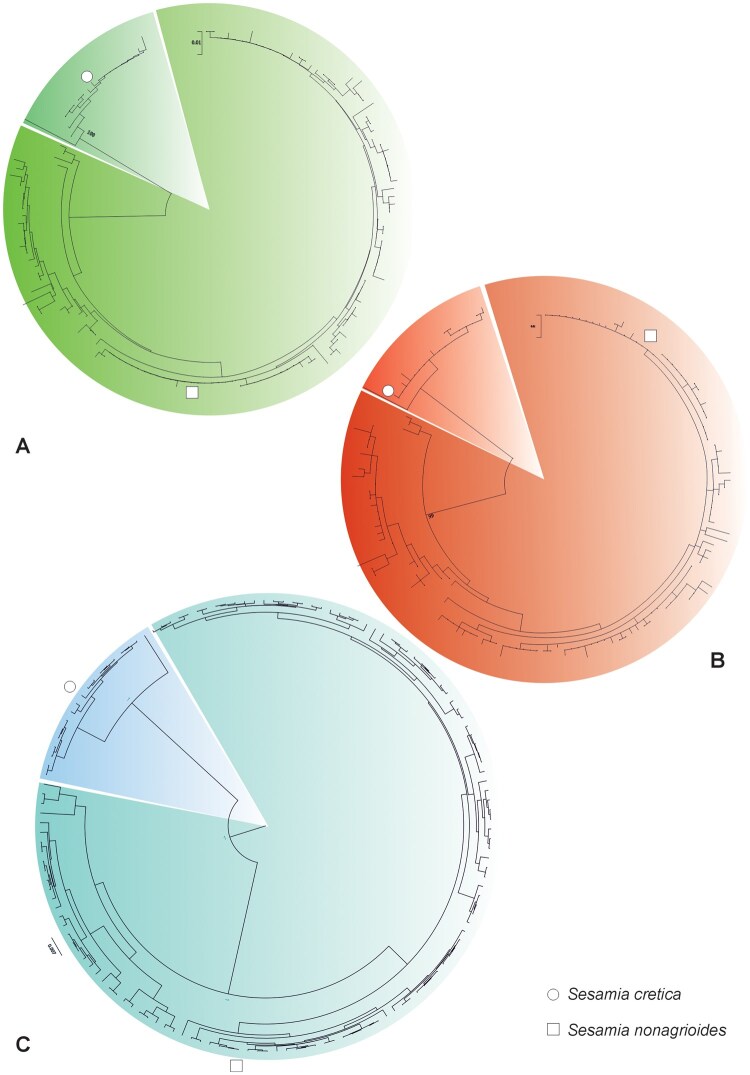
Phylogenetic relationships among taxa inferred using (A) Neighbor-Joining (NJ) and (B) Maximum Likelihood (ML) in MEGA12, and (C) Bayesian inference (BI) in BEAST, based on a 388 bp alignment of 146 sequences. Bootstrap values (NJ, ML) and posterior probabilities (BI) are shown above branches. Circles = S. cretica (this study), squares = S. nonagrioides (this study). Trees are unrooted due to short sequence length.

**Fig. 3.**
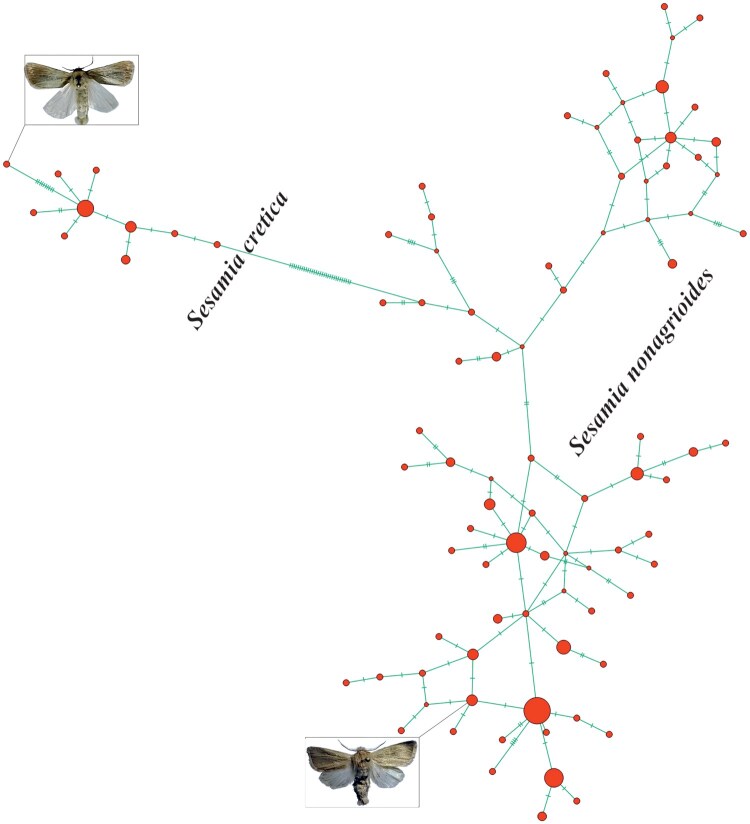
TCS haplotype network of Sesamia cretica and Sesamia nonagrioides. Each species forms a distinct cluster with no overlap. Sesamia cretica displays a compact and coherent network, while Sesamia nonagrioides shows a more dispersed pattern, reflecting greater genetic diversity. Circle sizes are proportional to haplotype frequency; green lines represent single nucleotide mutations between haplotypes.

Sequence-based phylogenetic analysis clearly separated *S. cretica* and *S. nonagrioides* with high bootstrap support (Fig. 2). Haplotype network analysis revealed a compact and uniform network for *S. cretica*, indicating low genetic diversity within this species, whereas *S. nonagrioides* exhibited a more dispersed and diverse haplotype structure (Fig. 3). Nucleotide diversity (π) was 0.00 for *S. cretica* and 0.01 for *S. nonagrioides*. The unusually low nucleotide diversity is likely influenced by the limited sample size (*n* = 50) and the fact that most samples were collected from a single province, potentially underestimating true genetic variation. A total of 10 unique haplotypes were identified for *S. cretica* and 67 for *S. nonagrioides*, reflecting a strong genetic differentiation between the 2 species. Phylogenetic reconstruction and haplotype network analyses (Figs. 2 and 3) confirmed the genetic separation of *S. cretica* and *S. nonagrioides*, fully consistent with morphological and M-PCR results. These analyses were used as validation tools rather than for population-level inference.

## Discussion

The larval stages of *S. cretica* and *S. nonagrioides* are significant pests of maize and sugarcane in Iran; however, their morphological similarity complicates accurate identification. In this study, we developed species-specific primers for M-PCR, enabling rapid and reliable identification of both species and preventing common misidentification errors. This approach aligns with studies using mitochondrial DNA markers, such as those investigating population structures in *Ectomyelois ceratoniae* Zeller (Malek Shahkouyi et al. 2019). Our method successfully enabled the rapid identification of these species, which is essential for IPM (Zhou et al. 2023, Hévin et al. 2024, Junqueira et al. 2024).

Previous taxonomic studies have revealed cryptic identities within the *S. nonagrioides* complex (Kergoat et al. 2015). These findings underscore the necessity of developing precise molecular techniques, such as M-PCR, to differentiate closely related species and enhance pest management efforts. Likewise, the complexity of the *S. cretica* group highlights the limitations of morphological identification alone, making it crucial to combine molecular and phylogenetic methods for accurate species delimitation (Le Ru et al. 2022).

Studies such as Gilligan et al. (2015) have demonstrated that molecular techniques, including Real-Time PCR, are significantly more effective than traditional morphological methods, especially for identifying immature stages. Similarly, Thomas et al. (2022) confirmed the limitations of larval-stage morphological identification and showed that molecular tools greatly enhance both the accuracy and speed of species diagnosis. This assay also complements the DNA barcoding approach used by Mehravar et al. (2017) for the same two species in Iran. While their study detected intraspecific variation using *COI* sequences, our M-PCR method provides faster, cost-effective species diagnosis without sequencing while maintaining high accuracy.

In this study, species-specific primers were designed based on gene sequences from diverse populations and validated using Primer-BLAST. Both single and M-PCR assays produced distinct bands for the target species, with no cross-reactivity observed with closely related taxa. These results were consistent with morphological and sequencing data (Andrews et al. 2022, Maggio et al. 2022). High reproducibility across technical and biological replicates, along with appropriate positive and negative controls, confirmed the method’s accuracy and reliability under both laboratory and field conditions.

Other studies have successfully applied M-PCR to distinguish closely related species and confirmed its robustness under varying experimental conditions (Hosseini-Chegeni et al. 2017, Kuwazaki et al. 2023). In this study, phylogenetic analysis clearly separated *S. cretica* and *S. nonagrioides*, supported by high bootstrap values. Haplotype network analysis further showed a uniform genetic structure in *S. cretica*, in contrast to the higher genetic diversity observed in *S. nonagrioides*.

These differences may reflect distinct population histories, levels of gene flow, or environmental adaptations (Nagoshi et al. 2006, Tay et al. 2022). Such patterns, particularly in *S. nonagrioides*, could also be consistent with the presence of cryptic lineages previously reported by Kergoat et al. (2015). Although haplotype diversity was higher in *S. nonagrioides* than in *S. cretica*, overall nucleotide diversity was low, likely due to limited geographic coverage and small sample size. Broader sampling across Iran and neighboring regions is needed to capture full genetic variation and clarify population structure. Although mitochondrial DNA markers cannot detect potential hybrids, no evidence of hybridization between *S. cretica* and *S. nonagrioides* has been reported. Previous molecular and ecological studies (Kergoat et al. 2015, Le Ru et al. 2022) indicate clear genetic boundaries between these species, supporting the reliability of mtDNA-based identification in this system.

While field trials were not conducted in this study, accurate early-stage identification could improve IPM outcomes by reducing unnecessary pesticide applications and enabling targeted interventions. Moreover, preliminary tests on a subset of damaged or partially degraded larval specimens (including additional samples from Lorestan Province) indicated that the assay can still produce reliable amplification when DNA quality is suboptimal, suggesting robustness for field-collected material. While the M-PCR method demonstrated high accuracy, further validation across larger and geographically diverse populations from Iran and neighboring countries is recommended. Future studies may also benefit from incorporating additional genetic markers or employing more advanced molecular techniques—such as digital PCR or real-time PCR—to further enhance diagnostic precision.

## Supplementary Material

ieaf105_Supplementary_Data
