# Supplementary material for: Multiplex PCR-based identification of two sympatric stem borer species, Sesamia cretica and Sesamia nonagrioides (Lepidoptera: Noctuidae)
Source: J Insect Sci. 2025 Dec 19;25(6):ieaf105. doi: 10.1093/jisesa/ieaf105 (PMC12715308; doi:10.1093/jisesa/ieaf105)
Supplement: ieaf105_Supplementary_Data [file ieaf105_supplementary_data.docx]

* 20 * 40 * 60 * 80 * 100 * 120 * 140 *
Sc-KU891975 TTATTACCCCCTTCTTTAACTTTATTAATTTCAAGAAGAATTGTAGAAAATGGAGCAGGTACAGGATGAACGGTATATCCCCCCCTCTCATCTAATATTGCTCATGGAGGAAGATCTGTAGATTTAGCTATTTTTTCCCTTCATTTAGCG:150
Sc-KU891973 TTATTACCCCCTTCTTTAACTTTATTAATTTCAAGAAGAATTGTAGAAAATGGAGCAGGTACCGGATGAACGGTATATCCCCCCCTCTCATCTAATATTGCTCATGGAGGAAGATCTGTAGATTTAGCTATTTTTTCCCTTCATTTAGCG:150
Sc-KU891972 TTATTACCCCCTTCTTTAACTTTATTAATTTCAAGAAGAATTGTAGAAAATGGAGCAGGTACAGGATGAACGGTATATCCCCCCCTCTCATCTAATATTGCTCATGGAGGAAGATCTGTAGATTTAGCTATTTTTTCCCTTCATTTAGCG:150
Sc-OM350099 TTATTACCCCCTTCTTTAACTTTATTAATTTCAAGAAGAATTGTAGAAAATGGAGCAGGTACAGGATGAACGGTATATCCCCCCCTCTCATCTAATATTGCTCATGGAGGAAGATCTGTAGATTTAGCTATTTTTTCCCTTCATTTAGCG:150
Sc-OM350097 TTATTACCTCCTTCTTTAACTTTATTAATTTCAAGAAGAATTGTAGAAAATGGAGCAGGTACAGGATGAACGGTATATCCCCCCCTCTCATCTAATATTGCTCATGGAGGAAGATCTGTAGATTTAGCTATTTTTTCCCTTCATTTAGCG:150
Sc-OM350096 TTATTACCCCCTTCTTTAACTTTATTAATTTCAAGAAGAATTGTAGAAAATGGAGCAGGTACAGGATGAACGGTATATCCCCCCCTCTCATCTAATATTGCTCATGGAGGAAGATCTGTAGATTTAGCTATTTTTTCCCTTCATTTAGCG:150
Sc-MH851121 TTATTACCCCCTTCTTTAACTTTATTAATTTCAAGAAGAATTGTAGAAAATGGAGCAGGTACAGGATGAACGGTATATCCCCCCCTCTCATCTAATATTGCTCATGGAGGAAGATCTGTAGATTTAGCTATTTTTTCCCTTCATTTAGCG:150
Sc-PV646697 TTATTACCCCCTTCTTTAACTTTATTAATTTCAAGAAGAATTGTAGAAAATGGAGCAGGTACAGGATGAACGGTATATCCCCCCCTCTCATCTAAAATTGCTCATGGAGGAAGATCTGTAGATTTAGCTATTTTTTCCCTTCATTTAGCG:150
Sc-KU891978 TTATTACCCCCTTCTTTAACTTTATTAATTTCAAGAAGAATTGTAGAAAATGGAGCAGGTACAGGATGAACGGTATATCCCCCCCTCTCATCTAATATTGCTCATGGAGGAAGATCTGTAGATTTAGCTATTTTTTCCCTTCATTTAGCG:150
Sc-KU891976 TTATTACCCCCTTCTTTAACTTTATTAATTTCAAGAAGAATTGTAGAAAATGGAGCAGGTACAGGATGAACGGTATATCCCCCCCTCTCATCTAATATTGCTCATGGAGGAAGATCTGTAGATTTAGCTATTTTTTCCCTTCATTTAGCG:150
Sc-KU891977 TTATTACCCCCTTCTTTAACTTTATTAATTTCAAGAAGAATTGTAGAAAATGGAGCAGGTACAGGATGAACGGTATATCCCCCCCTCTCATCTAATATTGCTCATGGAGGAAGATCTGTAGATTTAGCTATTTTTTCCCTTCATTTAGCG:150
Sc-OR758844 TTATTACCCCCTTCTTTAACTTTATTAATTTCAAGAAGAATTGTAGAAAATGGAGCAGGTACAGGATGAACGGTATATCCCCCCCTCTCATCTAATATTGCTCATGGAGGAAGATCTGTAGATTTAGCTATTTTTTCCCTTCATTTAGCG:150
Sc-GWOSA261-10 TTATTACCCCCTTCTTTAACTTTATTAATTTCAAGAAGAATTGTAGAAAATGGAGCAGGTACAGGATGAACGGTATATCCCCCCCTCTCATCTAATATTGCTCATGGAGGAAGATCTGTAGATTTAGCTATTTTTTCCCTTCATTTAGCG:150
Sc-GWOSA262-10 TTATTACCCCCTTCTTTAACTTTATTAATTTCAAGAAGAATTGTAGAAAATGGAGCAGGTACAGGATGAACGGTATATCCCCCCCTCTCATCTAATATTGCTCATGGAGGAAGATCTGTAGATTTAGCTATTTTTTCCCTTCATTTAGCG:150
Sc-GBMIN83467-17 TTATTACCCCCTTCTTTAACTTTATTAATTTCAAGAAGAATTGTAGAAAATGGAGCAGGTACAGGATGAACGGTATATCCCCCCCTCTCATCTAATATTGCTCATGGAGGAAGATCTGTAGATTTAGCTATTTTTTCCCTTCATTTAGCG:150
Sc-GBMIN83468-17 TTATTACCCCCTTCTTTAACTTTATTAATTTCAAGAAGAATTGTAGAAAATGGAGCAGGTACAGGATGAACGGTATATCCCCCCCTCTCATCTAATATTGCTCATGGAGGAAGATCTGTAGATTTAGCTATTTTTTCCCTTCATTTAGCG:150
Sc-GBGL20669-18 TTATTACCCCCTTCTTTAACTTTATTAATTTCAAGAAGAATTGTAGAAAATGGAGCAGGTACAGGATGAACGGTATATCCCCCCCTCTCATCTAATATTGCTCATGGAGGAAGATCTGTAGATTTAGCTATTTTTTCCCTTCATTTAGCG:150
Sc-GBGL20668-18 TTATTACCCCCTTCTTTAACTTTATTAATTTCAAGAAGAATTGTAGAAAATGGAGCAGGTACCGGATGAACGGTATATCCCCCCCTCTCATCTAATATTGCTCATGGAGGAAGATCTGTAGATTTAGCTATTTTTTCCCTTCATTTAGCG:150
Sc-LNAUY1377-19 TTATTACCCCCTTCTTTAACTTTATTAATTTCAAGAAGAATTGTAGAAAATGGAGCAGGTACAGGATGAACGGTATATCCCCCCCTCTCATCTAATATTGCTCATGGAGGAAGATCTGTAGATTTAGCTATTTTTTCCCTTCATTTAGCG:150
Sc-GBGL24680-19 TTATTACCCCCTTCTTTAACTTTATTAATTTCAAGAAGAATTGTAGAAAATGGAGCAGGTACAGGATGAACGGTATATCCCCCCCTCTCATCTAATATTGCTCATGGAGGAAGATCTGTAGATTTAGCTATTTTTTCCCTTCATTTAGCG:150
Sc-GBGL24681-19 TTATTACCCCCTTCTTTAACTTTATTAATTTCAAGAAGAATTGTAGAAAATGGAGCAGGTACAGGATGAACGGTATATCCCCCCCTCTCATCTAATATTGCTCATGGAGGAAGATCTGTAGATTTAGCTATTTTTTCCCTTCATTTAGCG:150
Sc-LNAUY1379-19 TTATTACCCCCTTCTTTAACTTTATTAATTTCAAGAAGAATTGTAGAAAATGGAGCAGGTACAGGATGAACGGTATATCCCCCCCTCTCATCTAATATTGCTCATGGAGGAAGATCTGTAGATTTAGCTATTTTTTCCCTTCATTTAGCG:150
Sc-GBGL24682-19 TTATTACCCCCTTCTTTAACTTTATTAATTTCAAGAAGAATTGTAGAAAATGGAGCAGGTACAGGATGAACGGTATATCCCCCCCTCTCATCTAATATTGCTCATGGAGGAAGATCTGTAGATTTAGCTATTTTTTCCCTTCATTTAGCG:150
Sc-LNAUY1378-19 TTATTACCCCCTTCTTTAACTTTATTAATTTCAAGAAGAATTGTAGAAAATGGAGCAGGTACAGGATGAACGGTATATCCCCCCCTCTCATCTAATATTGCTCATGGAGGAAGATCTGTAGATTTAGCTATTTTTTCCCTTCATTTAGCG:150
Sc-GBGL24687-19 TTATTACCCCCTTCTTTAACTTTATTAATTTCAAGAAGAATTGTAGAAAATGGAGCAGGTACAGGATGAACGGTATATCCCCCCCTCTCATCTAATATTGCTCATGGAGGAAGATCTGTAGATTTAGCTATTTTTTCCCTTCATTTAGCG:150
Sc-GBGL24686-19 TTATTACCCCCTTCTTTAACTTTATTAATTTCAAGAAGAATTGTAGAAAATGGAGCAGGTACAGGATGAACGGTATATCCCCCCCTCTCATCTAATATTGCTCATGGAGGAAGATCTGTAGATTTAGCTATTTTTTCCCTTCATTTAGCG:150
Sc-GBMNE61282-22 TTATTACCCCCTTCTTTAACTTTATTAATTTCAAGAAGAATTGTAGAAAATGGAGCAGGTACAGGATGAACGGTATATCCCCCCCTCTCATCTAATATTGCTCATGGAGGAAGATCTGTAGATTTAGCTATTTTTTCCCTTCATTTAGCG:150
Sc-GBMNE61283-22 TTATTACCTCCTTCTTTAACTTTATTAATTTCAAGAAGAATTGTAGAAAATGGAGCAGGTACAGGATGAACGGTATATCCCCCCCTCTCATCTAATATTGCTCATGGAGGAAGATCTGTAGATTTAGCTATTTTTTCCCTTCATTTAGCG:150
Sc-GBMNE61285-22 TTATTACCCCCTTCTTTAACTTTATTAATTTCAAGAAGAATTGTAGAAAATGGAGCAGGTACAGGATGAACGGTATATCCCCCCCTCTCATCTAATATTGCTCATGGAGGAAGATCTGTAGATTTAGCTATTTTTTCCCTTCATTTAGCG:150
Sc-GBAAW8767-24 TTATTACCCCCTTCTTTAACTTTATTAATTTCAAGAAGAATTGTAGAAAATGGAGCAGGTACAGGATGAACGGTATATCCCCCCCTCTCATCTAATATTGCTCATGGAGGAAGATCTGTAGATTTAGCTATTTTTTCCCTTCATTTAGCG:150
Sn-GBGL12313-13 CTATTACCACCATCCTTAACCCTTTTAATTTCAAGTAGAATTGTAGAAAATGGGGCTGGAACAGGATGAACAGTTTATCCCCCACTTTCATCTAACATCGCTCATGGAGGGAGATCTGTAGATTTAGCTATTTTTTCCCTTCATTTAGCT:150
Sn-JF274189 CTATTACCACCATCCTTAACCCTTTTAATTTCAAGTAGAATTGTAGAAAATGGGGCTGGAACAGGATGAACAGTTTACCCCCCACTTTCATCTAACATCGCTCATGGAGGGAGATCTGTAGATTTAGCTATTTTTTCCCTTCATTTAGCT:150
Sn-JF274179 CTATTACCACCATCCTTAACCCTTTTAATTTCAAGTAGAATTGTAGAAAATGGGGCTGGAACAGGATGAACAGTTTACCCCCCACTTTCATCTAACATCGCTCATGGAGGAAGATCTGTAGATTTAGCTATTTTTTCCCTTCATTTAGCT:150
Sn-JF274178 CTATTACCACCATCCTTAACCCTTTTAATTTCAAGTAGAATTGTAGAAAATGGGGCTGGAACAGGATGAACAGTTTACCCCCCACTTTCATCTAACATCGCTCATGGAGGAAGATCTGTAGATTTAGCTATTTTTTCCCTTCATTTAGCT:150
Sn-JF274177 CTATTACCACCATCCTTAACCCTTTTAATTTCAAGTAGAATTGTAGAAAATGGGGCTGGAACAGGATGAACAGTTTACCCCCCACTTTCATCTAACATCGCTCATGGAGGAAGATCTGTAGATTTAGCTATTTTTTCCCTTCATTTAGCT:150
Sn-JF274173 CTATTACCACCATCCTTAACCCTTTTAATTTCAAGTAGAATTGTAGAAAATGGGGCTGGAACAGGATGAACAGTTTACCCCCCACTTTCATCTAACATCGCTCATGGAGGAAGATCTGTAGATTTAGCTATTTTTTCCCTTCATTTAGCT:150
Sn-JF274201 CTATTACCACCATCCTTAACCCTTTTAATTTCAAGTAGAATTGTAGAAAATGGGGCTGGAACAGGATGAACAGTTTACCCCCCACTTTCATCTAACATCGCTCATGGGGGAAGATCTGTAGATTTAGCTATTTTTTCCCTTCATTTAGCT:150
Sn-JF274203 CTATTACCACCATCCTTAACCCTTTTAATTTCAAGTAGAATTGTAGAAAATGGGGCTGGAACAGGATGAACAGTTTACCCCCCACTTTCATCTAACATCGCTCATGGAGGAAGATCTGTAGATTTAGCTATTTTTTCCCTTCATTTAGCT:150
Sn-JF274199 CTATTACCACCATCCTTAACCCTTTTAATTTCAAGTAGAATTGTAGAAAATGGGGCTGGAACAGGATGAACAGTTTACCCCCCACTTTCATCTAACATCGCTCATGGAGGAAGATCTGTAGATTTAGCTATTTTTTCCCTTCATTTAGCT:150
Sn-JF274205 CTATTACCACCATCCTTAACCCTTTTAATTTCAAGTAGAATTGTAGAAAATGGAGCTGGAACAGGATGAACAGTTTACCCCCCACTTTCATCTAATATTGCTCATGGAGGAAGATCTGTAGATTTAGCTATTTTTTCCCTTCATTTAGCT:150
Sn-JF274204 CTATTACCACCATCCTTAACCCTTTTAATTTCAAGTAGAATTGTAGAAAATGGAGCTGGAACAGGATGAACAGTTTACCCCCCACTTTCATCTAATATTGCTCATGGAGGAAGATCTGTAGATTTAGCTATTTTTTCCCTTCATTTAGCT:150
Sn-JF274192 CTATTACCACCATCCTTAACCCTTTTAATTTCAAGTAGAATTGTAGAAAATGGGGCTGGAACAGGATGAACAGTTTACCCCCCACTTTCATCTAACATCGCTCATGGAGGAAGATCTGTAGATTTAGCTATTTTTTCCCTTCATTTAGCT:150
Sn-JF274181 CTATTACCACCATCCTTAACCCTTTTAATTTCAAGTAGAATTGTAGAAAATGGGGCTGGAACAGGATGAACAGTTTACCCCCCACTTTCATCTAACATCGCTCATGGAGGAAGATCTGTAGATTTAGCTATTTTTTCCCTTCATTTAGCT:150
Sn-JF274198 CTATTACCACCATCCTTAACCCTTTTAATTTCAAGTAGAATTGTAGAAAATGGGGCTGGAACAGGATGAACAGTTTACCCCCCACTTTCATCTAACATCGCTCATGGAGGAAGATCTGTAGATTTAGCTATTTTTTCCCTTCATTTAGCT:150
Sn-JF274195 CTATTACCACCATCCTTAACCCTTTTAATTTCAAGTAGAATTGTAGAAAATGGGGCTGGAACAGGATGAACAGTTTACCCCCCACTTTCATCTAACATCGCTCATGGAGGAAGATCTGTAGATTTAGCTATTTTTTCCCTTCATTTAGCT:150
Sn-AJ829718 CTATTACCACCATCCTTAACCCTTTTAATTTCAAGTAGAATTGTAGAAAATGGGGCTGGAACAGGATGAACAGTTTACCCCCCACTTTCATCTAACATCGCTCACGGAGGAAGATCTGTAGATTTAGCTATTTTTTCCCTTCATTTAGCT:150
Sn-AY649322 CTATTACCACCATCCTTAACCCTTTTAATTTCAAGTAGAATTGTAGAAAATGGGGCTGGAACAGGATGAACAGTTTACCCCCCACTTTCATCTAACATCGCTCACGGAGGAAGATCTGTAGATTTAGCTATTTTTTCCCTTCATTTAGCT:150
Sn-JF274196 CTATTACCACCATCCTTAACCCTTTTAATTTCAAGTAGAATTGTAGAAAATGGGGCTGGAACAGGATGAACAGTTTACCCCCCACTTTCATCTAACATCGCTCATGGAGGAAGATCTGTAGATTTAGCTATTTTTTCCCTTCATTTAGCT:150
Sn-JF274193 CTATTACCACCATCCTTAACCCTTTTAATTTCAAGTAGAATTGTAGAAAATGGGGCTGGAACAGGATGAACAGTTTACCCCCCACTTTCATCTAACATCGCTCATGGAGGAAGATCTGTAGATTTAGCTATTTTTTCCCTTCATTTAGCT:150
Sn-JF274184 CTATTACCACCATCCTTAACCCTTTTAATTTCAAGTAGAATTGTAGAAAATGGGGCTGGAACAGGATGAACAGTTTACCCCCCACTTTCATCTAACATCGCTCATGGAGGAAGATCTGTAGATTTAGCTATTTTTTCCCTTCATTTAGCT:150
Sn-JF274191 CTATTACCACCATCCTTAACCCTTTTAATTTCAAGTAGAATTGTAGAAAATGGGGCTGGAACAGGATGAACAGTTTACCCCCCACTTTCATCTAACATCGCTCATGGAGGGAGATCTGTAGATTTAGCTATTTTTTCCCTTCATTTAGCT:150
Sn-JF274183 CTATTACCACCATCCTTAACCCTTTTAATTTCAAGTAGAATTGTAGAAAATGGGGCTGGAACAGGATGAACAGTTTACCCCCCACTTTCATCTAACATCGCTCATGGAGGAAGATCTGTAGATTTAGCTATTTTTTCCCTTCATTTAGCT:150
Sn-JF274169 CTATTACCACCATCCTTAACCCTTTTAATTTCAAGTAGAATTGTAGAAAATGGGGCTGGAACAGGATGAACAGTTTACCCCCCACTTTCATCTAACATCGCTCACGGAGGAAGATCTGTAGATTTAGCTATTTTTTCCCTTCATTTAGCT:150
Sn-F274188 CTATTACCACCATCCTTAACCCTTTTAATTTCAAGTAGAATTGTAGAAAATGGGGCTGGAACAGGATGAACAGTTTACCCTCCACTTTCATCTAACATCGCTCATGGGGGAAGATCTGTAGATTTAGCTATTTTTTCCCTTCATTTAGCT:150
Sn-JF274186 CTATTACCACCATCCTTAACCCTTTTAATTTCAAGTAGAATTGTAGAAAATGGGGCTGGAACAGGGTGAACAGTTTACCCCCCACTTTCATCTAACATCGCTCATGGAGGAAGATCTGTAGATTTAGCTATTTTTTCCCTTCATTTAGCT:150
Sn-JF274176 CTATTACCACCATCCTTAACCCTTTTAATTTCAAGTAGAATTGTAGAAAATGGGGCTGGAACAGGATGAACAGTTTACCCCCCACTTTCATCTAACATCGCTCATGGAGGAAGATCTGTAGATTTAGCTATTTTTTCCCTTCATTTAGCT:150
Sn-AJ829716 CTATTACCACCATCCTTAACCCTTTTAATTTCAAGTAGAATTGTAGAAAATGGGGCTGGAACAGGATGAACAGTTTACCCCCCACTTTCATCTAACATCGCTCATGGAGGAAGATCTGTAGATTTAGCTATTTTTTCCCTTCATTTAGCT:150
Sn-JF274175 CTATTACCACCATCCTTAACCCTTTTAATTTCAAGTAAAATTGTAGAAAATGGAGCTGGAACAGGATGAACAGTTTACCCCCCACTTTCATCTAACATCGCTCATGGAGGAAGATCTGTAGATTTAGCTATTTTTTCCCTTCATTTAGCT:150
Sn-JF274170 CTATTACCACCATCCTTAACCCTTTTAATTTCAAGTAGAATTGTAGAAAATGGGGCTGGAACAGGATGAACAGTTTACCCCCCACTTTCATCTAACATCGCTCACGGAGGAAGATCTGTAGATTTAGCTATTTTTTCCCTTCATTTAGCT:150
Sn-AJ829715 CTATTACCACCATCCTTAACCCTTTTAATTTCAAGTAGAATTGTAGAAAATGGGGCTGGAACAGGATGAACAGTTTACCCCCCACTTTCATCTAACATCGCTCATGGAGGAAGATCTGTAGATTTAGCTATTTTTTCCCTTCATTTAGCT:150
Sn-JF274182 CTATTACCACCATCCTTAACCCTTTTAATTTCAAGTAGAATTGTAGAAAATGGGGCTGGAACAGGATGAACAGTTTACCCCCCTCTTTCATCTAACATCGCTCATGGAGGAAGATCTGTAGATTTAGCTATTTTTTCCCTTCATTTAGCT:150
Sn-JF274194 CTATTACCACCATCCTTAACCCTTTTAATTTCAAGTAGAATTGTAGAAAATGGGGCTGGAACAGGATGAACAGTTTACCCCCCACTTTCATCTAACATCGCTCATGGAGGAAGATCTGTAGATTTAGCTATTTTTTCCCTTCATTTAGCT:150
Sn-JF274202 CTATTACCACCATCCTTAACCCTTTTAATTTCAAGTAGAATTGTAGAAAATGGAGCTGGAACAGGATGAACAGTTTACCCCCCACTTTCATCTAACATCGCTCATGGGGGAAGATCTGTAGATTTAGCTATTTTTTCCCTTCATTTGGCT:150
Sn-JF274187 CTATTACCACCATCCTTAACCCTTTTAATTTCAAGTAGAATTGTAGAAAATGGGGCTGGAACAGGATGAACAGTTTACCCCCCACTTTCATCTAACATCGCTCATGGAGGAAGATCTGTAGATTTAGCTATTTTTTCCCTTCATTTAGCT:150
Sn-JF274172 CTATTACCACCATCCTTAACCCTTTTAATTTCAAGTAGAATTGTAGAAAATGGGGCTGGAACAGGATGAACAGTTTACCCCCCACTTTCATCTAACATCGCTCATGGAGGAAGATCTGTAGATTTAGCTATTTTTTCCCTTCATTTAGCT:150
Sn-JF274168 CTATTACCACCATCCTTAACCCTTTTAATTTCAAGTAGAATTGTAGAAAATGGGGCTGGAACAGGATGAACAGTTTACCCCCCACTTTCATCTAACATCGCTCACGGAGGAAGATCTGTAGATTTAGCTATTTTTTCCCTTCATTTAGCT:150
Sn-JF274197 CTATTACCACCATCCTTAACCCTTTTAATTTCAAGTAGAATTGTAGAAAATGGGGCTGGAACAGGATGAACAGTTTACCCCCCACTTTCATCTAACATCGCTCATGGAGGAAGATCTGTAGATTTAGCTATTTTTTCCCTTCATTTAGCT:150
Sn-JF274185 CTATTACCACCATCCTTAACCCTTTTAATTTCAAGTAGAATTGTAGAAAATGGGGCTGGAACAGGATGAACAGTTTACCCTCCACTTTCATCTAACATCGCTCATGGGGGAAGATCTGTAGATTTAGCTATTTTTTCCCTTCATTTAGCT:150
Sn-JF274200 CTATTACCACCATCCTTAACCCTTTTAATTTCAAGTAGAATTGTAGAAAATGGGGCTGGAACAGGATGAACAGTTTACCCCCCACTTTCATCTAACATCGCTCATGGAGGAAGATCTGTAGATTTAGCTATTTTTTCCCTTCATTTAGCT:150
Sn-JF274180 CTATTACCACCATCCTTAACCCTTTTAATTTCAAGTAGAATTGTAGAAAATGGGGCTGGAACAGGATGAACAGTTTACCCCCCTCTTTCATCTAACATCGCTCATGGAGGAAGATCTGTAGATTTAGCTATTTTTTCCCTTCATTTAGCT:150
Sn-JF274171 CTATTACCACCATCCTTAACCCTTTTAATTTCAAGTAGAATTGTAGAAAATGGGGCTGGAACAGGATGAACAGTTTACCCCCCACTTTCATCTAACATCGCTCACGGAGGAAGATCTGTAGATTTAGCTATTTTTTCCCTTCATTTAGCT:150
Sn-AJ829717 CTATTACCACCATCCTTAACCCTTTTAATTTCAAGTAGAATTGTAGAAAATGGGGCTGGAACAGGATGAACAGTTTACCCCCCACTTTCATCTAACATCGCTCATGGAGGAAGATCTGTAGATTTAGCTATTTTTTCCCTTCATTTAGCT:150
Sn-JF274174 CTATTACCACCATCCTTAACCCTTTTAATTTCAAGTAGAATTGTAGAAAATGGGGCTGGAACAGGATGAACAGTTTACCCCCCACTTTCATCTAACATCGCTCATGGAGGAAGATCTGTAGATTTAGCTATTTTTTCCCTTCATTTAGCT:150
Si-KP658205 TTATTACCCCCCTCTTTAACTCTTTTAATTTCAAGTAGAATTGTAGAAAATGGAGCAGGAACTGGATGAACAGTGTACCCCCCACTTTCATCTAATATTGCTCATGGAGGAAGATCAGTAGATCTAGCTATTTTTTCCCTTCATTTAGCT:150
Si-MG838445 TTATTACCCCCCTCTTTAACTCTTTTAATTTCAAGTAGAATTGTAGAAAATGGAGCAGGAACTGGATGAACAGTGTACCCCCCACTTTCATCTAATATTGCTCATGGAGGAAGATCAGTGGATCTAGCTATTTTTTCCCTTCATTTAGCT:150
Si-MT775880 TTATTACCCCCCTCTTTAACTCTTTTAATTTCAAGCAGAATTGTAGAAAATGGAGTAGGAACTGGATGAACAGTATACCCCCCACTTTCATCTAATATTGCTCATGGAGGAAGATCAGTAGATTTAGCTATTTTCTCCCTTCATTTAGCT:150
Si-MT734563 TTATTACCCCCCTCTTTAACTCTTTTAATTTCAAGCAGAATTGTAGAAAATGGAGTAGGAACTGGATGAACAGTATACCCCCCACTTTCATCTAATATTGCTCATGGAGGAAGATCAGTAGATTTAGCTATTTTCTCCCTTCATTTAGCT:150
Si-MT124087 TTATTACCCCCCTCTTTAACTCTTTTAATTTCAAGTAGAATTGTAGAAAATGGAGCAGGAACTGGATGAACAGTGTACCCCCCACTTTCATCTAATATTGCTCATGGAGGAAGATCAGTAGATCTAGCTATTTTTTCCCTTCATTTAGCT:150
Si-MT124086 TTATTACCCCCCTCTTTAACTCTTTTAATTTCAAGTAGAATTGTAGAAAATGGAGCAGGAACTGGATGAACAGTGTACCCCCCACTTTCATCTAATATTGCTCATGGAGGAAGATCAGTAGATCTAGCTATTTTTTCCCTTCATTTAGCT:150
Si-KT250763 TTATTACCCCCCTCTTTAACTCTTTTAATTTCAAGTAGAATTGTAGAAAATGGAGCAGGAACTGGATGAACAGTGTACCCCCCACTTTCATCTAATATTGCTCATGGAGGAAGATCAGTAGATCTAGCTATTTTTTCCCTTCATTTAGCT:150
Si-GU681997 TTATTACCCCCCTCTTTAACTCTTTTAATTTCAAGTAGAATTGTAGAAAATGGAGCAGGAACTGGATGAACAGTGTACCCCCCACTTTCATCTAATATTGCTCATGGAGGAAGATCAGTAGATCTAGCTATTTTTTCCCTTCATTTAGCT:150
Si-MT734554 TTATTACCCCCCTCTTTAACTCTTTTAATTTCAAGTAGAATTGTAGAAAATGGAGCAGGAACTGGATGAACAGTATACCCCCCACTTTCATCTAATATTGCCCATGGAGGAAGATCAGTAGATTTAGCTATTTTCTCCCTTCATTTAGCT:150
Si-MT734546 TTATTACCCCCCTCTTTAACTCTTTTAATTTCAAGCAGAATTGTAGAAAATGGAGTAGGAACTGGATGAACAGTGTACCCCCCACTTTCATCTAATATTGCTCATGGAGGAAGATCAGTAGATTTAGCCATTTTCTCCCTTCATTTAGCT:150
Sca-MH851084 TTATTACCCCCATCCTTAACCCTTTTAATTTCAAGTAGAATTGTAGAAAACGGAGCAGGAACAGGATGAACAGTATACCCCCCACTTTCATCTAATATTGCTCATGGAGGAAGATCAGTAGATTTAGCTATTTTTTCTCTTCACTTAGCT:150
Sca-OR596544 TTATTACCCCCATCCTTAACCCTTTTAATTTCAAGTAGAATTGTAGAAAACGGAGCAGGAACAGGATGAACAGTATACCCCCCACTTTCATCTAATATTGCTCATGGAGGAAGATCAGTAGATTTAGCTATTTTTTCTCTTCACTTAGCT:150
Sca-OR596545 TTATTACCCCCATCCTTAACCCTTTTAATTTCAAGTAGAATTGTAGAAAACGGAGCAGGAACAGGATGAACAGTATACCCCCCACTTTCATCTAATATTGCTCATGGAGGAAGATCAGTAGATTTAGCTATTTTTTCTCTTCACTTAGCT:150
Sca-MH851102 TTATTACCCCCATCCTTAACCCTTTTAATTTCAAGTAGAATTGTAGAAAACGGAGCAGGAACAGGATGAACAGTATACCCCCCACTTTCATCTAATATTGCTCATGGAGGAAGATCAGTAGATTTAGCTATTTTTTCTCTTCACTTAGCT:150
Sca-KP682634 TTATTACCCCCATCCTTAACCCTTTTAATTTCAAGTAGAATCGTAGAAAACGGAGCAGGAACAGGATGAACAGTATATCCCCCACTTTCATCTAATATTGCTCATGGAGGAAGATCAGTAGATTTAGCTATTTTTTCTCTTCATTTAGCT:150
Sca-MK566706 TTATTACCCCCATCCTTAACCCTTTTAATTTCAAGTAGAATCGTAGAAAACGGAGCAGGAACAGGATGAACAGTATATCCCCCACTTTCATCTAATATTGCTCATGGGGGAAGATCAGTAGATTTAGCTATTTTTTCTCTTCATTTAGCT:150
Sca-MH851092 TTATTACCCCCATCCTTAACCCTTTTAATTTCAAGTAGAATCGTAGAAAACGGAGCAGGAACAGGATGAACAGTATATCCCCCACTTTCATCTAATATTGCTCATGGGGGAAGATCAGTAGATTTAGCTATTTTTTCTCTTCATTTAGCT:150
Sca-KF972231 TTATTACCCCCATCCTTAACCCTTTTAATTTCAAGTAGAATCGTAGAAAACGGAGCAGGAACAGGATGAACAGTATATCCCCCACTTTCATCTAATATTGCTCATGGGGGAAGATCAGTAGATTTAGCTATTTTTTCTCTTCATTTAGCT:150
Sca-MH851091 TTATTACCCCCATCCTTAACCCTTTTAATTTCAAGTAGAATTGTAGAAAACGGAGCAGGAACAGGATGAACAGTATACCCCCCACTTTCATCTAATATTGCTCATGGAGGAAGATCAGTAGATTTAGCTATTTTTTCTCTTCACTTAGCT:150
Sca-MH851099 TTATTACCCCCATCCTTAACCCTTTTAATTTCAAGTAGAATTGTAGAAAATGGAGCAGGAACAGGATGAACAGTATACCCCCCACTTTCATCTAATATTGCTCATGGGGGAAGATCAGTAGATTTAGCTATTTTTTCTCTTCACTTAGCT:150
Sa-MH851080 CTTCTTCCCCCCTCATTAACCTTATTAATTTCAAGAAGAATCGTAGAAAATGGAGCTGGAACTGGATGAACAGTGTACCCCCCACTTTCATCCAACATTGCACATGGGGGAAGATCAGTAGATTTAGCTATCTTCTCCCTCCACTTAGCT:150
Sa-MH851079 CTTCTTCCCCCCTCATTAACCTTATTAATTTCAAGAAGAATCGTAGAAAATGGAGCTGGAACTGGATGAACAGTGTACCCCCCACTTTCATCCAACATTGCACATGGGGGAAGATCAGTAGATTTAGCTATCTTCTCCCTCCACTTAGCT:150


 160 * 180 * 200 * 220 * 240 * 260 * 280 * 300
Sc-KU891975 GGTATTTCATCTATTTTAGGAGCTATTAATTTTATTACAACAATTATTAATATACGATTAAATAACTTATCTTTTGATCAAATACCTTTATTTGTTTGAGCTGTTGGAATTACTGCATTCTTATTATTATTATCTTTACCTGTTTTAGCA:300
Sc-KU891973 GGTATTTCATCTATTTTAGGAGCGATTAATTTTATTACAACAATTATTAATATACGATTAAATAACTTATCTTTTGATCAAATACCTTTATTTGTTTGAGCTGTTGGAATTACTGCATTCTTATTATTATTATCTTTACCTGTTTTAGCA:300
Sc-KU891972 GGTATTTCATCTATTTTAGGAGCTATTAATTTTATTACAACAATTATTAATATACGATTAAATAACTTATCTTTTGATCAAATACCTTTATTTGTTTGAGCTGTTGGAATTACTGCATTCTTATTATTATTATCTTTACCTGTTTTAGCA:300
Sc-OM350099 GGTATTTCATCTATTTTAGGAGCTATTAATTTTATTACAACAATTATTAATATACGATTAAATAACTTATCTTTTGATCAAATACCTTTATTTGTTTGAGCTGTTGGAATTACTGCATTCTTATTATTATTATCTTTACCTGTTTTAGCA:300
Sc-OM350097 GGTATTTCATCTATTTTAGGAGCTATTAATTTTATTACAACAATTATTAATATACGATTAAATAACTTATCTTTTGATCAAATACCTTTATTTGTTTGAGCTGTTGGAATTACTGCATTCTTATTATTATTATCTTTACCTGTTTTAGCA:300
Sc-OM350096 GGTATTTCATCTATTTTAGGAGCTATTAATTTTATTACAACAATTATTAATATACGATTAAATAACTTATCTTTTGATCAAATACCTTTATTTGTTTGAGCTGTTGGAATTACTGCATTCTTATTATTATTATCTTTACCTGTTTTAGCA:300
Sc-MH851121 GGTATTTCATCTATTTTAGGAGCTATTAATTTTATTACAACAATTATTAATATACGATTAAATAACTTATCTTTTGATCAAATACCTTTATTTGTTTGAGCTGTTGGAATTACTGCATTCTTATTATTATTATCTTTACCTGTTTTAGCA:300
Sc-PV646697 GGTATTTCATCTATTTTAGGAGCTATTAATTTTATTACAACAATTATTAATATACGATTAAATAACTTATCTTTTGATCAAATACCTTTATTTGTTTGAGCTGTTGGAATTACTGCATTCTTATTATTATTATCTTTACCTGTTTTAGCA:300
Sc-KU891978 GGTATTTCATCTATTTTAGGAGCTATTAATTTTATTACAACAATTATTAATATACGATTAAATAACTTATCTTTTGATCAAATACCTTTATTTGTTTGAGCTGTTGGAATTACTGCATTCTTATTATTATTATCTTTACCTGTTTTAGCA:300
Sc-KU891976 GGTATTTCATCTATTTTAGGAGCTATTAATTTTATTACAACAATTATTAATATACGATTAAATAACTTATCTTTTGATCAAATACCTTTATTTGTTTGAGCTGTTGGAATTACTGCATTCTTATTATTATTATCTTTACCTGTTTTAGCA:300
Sc-KU891977 GGTATTTCATCTATTTTAGGAACTATTAATTTTATTACAACAATTATTAATATACGATTAAATAACTTATCTTTTGATCAAATACCTTTATTTGTTTGAGCTGTTGGAATTACTGCATTCTTATTATTATTATCTTTACCTGTTTTAGCA:300
Sc-OR758844 GGTATTTCATCTATTTTAGGAACTATTAATTTTATTACAACAATTATTAATATACGATTAAATAACTTATCTTTTGATCAAATACCTTTATTTGTTTGAGCTGTTGGAATTACTGCATTCTTATTATTATTATCTTTACCTGTTTTAGCA:300
Sc-GWOSA261-10 GGTATTTCATCTATTTTAGGAGCTATTAATTTTATTACAACAATTATTAATATACGATTAAATAACTTATCTTTTGATCAAATACCTTTATTTGTTTGAGCTGTTGGAATTACTGCATTCTTATTATTATTATCTTTACCTGTTTTAGCA:300
Sc-GWOSA262-10 GGTATTTCATCTATTTTAGGAGCTATTAATTTTATTACAACAATTATTAATATACGATTAAATAACTTATCTTTTGATCAAATACCTTTATTTGTTTGAGCTGTTGGAATTACTGCATTCTTATTATTATTATCTTTACCTGTTTTAGCA:300
Sc-GBMIN83467-17 GGTATTTCATCTATTTTAGGAGCTATTAATTTTATTACAACAATTATTAATATACGATTAAATAACTTATCTTTTGATCAAATACCTTTATTTGTTTGAGCTGTTGGAATTACTGCATTCTTATTATTATTATCTTTACCTGTTTTAGCA:300
Sc-GBMIN83468-17 GGTATTTCATCTATTTTAGGAGCTATTAATTTTATTACAACAATTATTAATATACGATTAAATAACTTATCTTTTGATCAAATACCTTTATTTGTTTGAGCTGTTGGAATTACTGCATTCTTATTATTATTATCTTTACCTGTTTTAGCA:300
Sc-GBGL20669-18 GGTATTTCATCTATTTTAGGAGCTATTAATTTTATTACAACAATTATTAATATACGATTAAATAACTTATCTTTTGATCAAATACCTTTATTTGTTTGAGCTGTTGGAATTACTGCATTCTTATTATTATTATCTTTACCTGTTTTAGCA:300
Sc-GBGL20668-18 GGTATTTCATCTATTTTAGGAGCGATTAATTTTATTACAACAATTATTAATATACGATTAAATAACTTATCTTTTGATCAAATACCTTTATTTGTTTGAGCTGTTGGAATTACTGCATTCTTATTATTATTATCTTTACCTGTTTTAGCA:300
Sc-LNAUY1377-19 GGTATTTCATCTATTTTAGGAGCTATTAATTTTATTACAACAATTATTAATATACGATTAAATAACTTATCTTTTGATCAAATACCTTTATTTGTTTGAGCTGTTGGAATTACTGCATTCTTATTATTATTATCTTTACCTGTTTTAGCA:300
Sc-GBGL24680-19 GGTATTTCATCTATTTTAGGAGCTATTAATTTTATTACAACAATTATTAATATACGATTAAATAACTTATCTTTTGATCAAATACCTTTATTTGTTTGAGCTGTTGGAATTACTGCATTCTTATTATTATTATCTTTACCTGTTTTAGCA:300
Sc-GBGL24681-19 GGTATTTCATCTATTTTAGGAGCTATTAATTTTATTACAACAATTATTAATATACGATTAAATAACTTATCTTTTGATCAAATACCTTTATTTGTTTGAGCTGTTGGAATTACTGCATTCTTATTATTATTATCTTTACCTGTTTTAGCA:300
Sc-LNAUY1379-19 GGTATTTCATCTATTTTAGGAGCTATTAATTTTATTACAACAATTATTAATATACGATTAAATAACTTATCTTTTGATCAAATACCTTTATTTGTTTGAGCTGTTGGAATTACTGCATTCTTATTATTATTATCTTTACCTGTTTTAGCA:300
Sc-GBGL24682-19 GGTATTTCATCTATTTTAGGAGCTATTAATTTTATTACAACAATTATTAATATACGATTAAATAACTTATCTTTTGATCAAATACCTTTATTTGTTTGAGCTGTTGGAATTACTGCATTCTTATTATTATTATCTTTACCTGTTTTAGCA:300
Sc-LNAUY1378-19 GGTATTTCATCTATTTTAGGAGCTATTAATTTTATTACAACAATTATTAATATACGATTAAATAACTTATCTTTTGATCAAATACCTTTATTTGTTTGAGCTGTTGGAATTACTGCATTCTTATTATTATTATCTTTACCTGTTTTAGCA:300
Sc-GBGL24687-19 GGTATTTCATCTATTTTAGGAGCTATTAATTTTATTACAACAATTATTAATATACGATTAAATAACTTATCTTTTGATCAAATACCTTTATTTGTTTGAGCTGTTGGAATTACTGCATTCTTATTATTATTATCTTTACCTGTTTTAGCA:300
Sc-GBGL24686-19 GGTATTTCATCTATTTTAGGAACTATTAATTTTATTACAACAATTATTAATATACGATTAAATAACTTATCTTTTGATCAAATACCTTTATTTGTTTGAGCTGTTGGAATTACTGCATTCTTATTATTATTATCTTTACCTGTTTTAGCA:300
Sc-GBMNE61282-22 GGTATTTCATCTATTTTAGGAGCTATTAATTTTATTACAACAATTATTAATATACGATTAAATAACTTATCTTTTGATCAAATACCTTTATTTGTTTGAGCTGTTGGAATTACTGCATTCTTATTATTATTATCTTTACCTGTTTTAGCA:300
Sc-GBMNE61283-22 GGTATTTCATCTATTTTAGGAGCTATTAATTTTATTACAACAATTATTAATATACGATTAAATAACTTATCTTTTGATCAAATACCTTTATTTGTTTGAGCTGTTGGAATTACTGCATTCTTATTATTATTATCTTTACCTGTTTTAGCA:300
Sc-GBMNE61285-22 GGTATTTCATCTATTTTAGGAGCTATTAATTTTATTACAACAATTATTAATATACGATTAAATAACTTATCTTTTGATCAAATACCTTTATTTGTTTGAGCTGTTGGAATTACTGCATTCTTATTATTATTATCTTTACCTGTTTTAGCA:300
Sc-GBAAW8767-24 GGTATTTCATCTATTTTAGGAACTATTAATTTTATTACAACAATTATTAATATACGATTAAATAACTTATCTTTTGATCAAATACCTTTATTTGTTTGAGCTGTTGGAATTACTGCATTCTTATTATTATTATCTTTACCTGTTTTAGCA:300
Sn-GBGL12313-13 GGGATTTCATCTATTCTAGGAGCTATTAATTTTATTACAACAATTATTAATATACGATTAAATAATTTATCATTTGATCAAATACCATTATTTATTTGAGCTGTTGGAATTACTGCTTTTTTATTACTATTATCATTACCCGTTTTAGCA:300
Sn-JF274189 GGAATTTCATCTATTCTAGGAGCTATTAATTTTATTACAACAATTATTAATATACGATTAAATAATTTATCATTTGATCAAATACCATTATTTATTTGAGCTGTTGGAATTACTGCTTTTTTATTACTATTATCATTACCCGTTTTAGCA:300
Sn-JF274179 GGAATTTCATCTATTCTAGGAGCTATTAATTTTATTACAACAATTATTAATATACAATTAAATAATTTATCATTTGATCAAATACCATTATTTATTTGAGCTGTTGGGATTACTGCTTTTTTATTACTATTATCATTACCCGTTTTAGCA:300
Sn-JF274178 GGAATTTCATCTATTCTAGGAGCTATTAATTTTATTACAACAATTATTAATATACGATTAAATAATTTATCATTTGATCAAATACCATTATTTATTTGAGCTGTTGGAATTACTGCTTTTTTATTACTATTATCATTACCCGTTTTAGCA:300
Sn-JF274177 GGAATTTCATCTATTCTAGGAGCTATTAATTTTATTACAACAATTATTAATATACGATTAAATAATTTATCATTTGATCAAATACCATTATTTATTTGAGCTGTTGGGATTACTGCTTTTTTATTACTATTATCATTACCCGTTTTAGCA:300
Sn-JF274173 GGAATTTCATCTATTCTAGGAGCTATTAATTTTATTACAACAATTATTAATATACGATTAAATAATTTATCATTTGATCAAATACCATTATTTATTTGAGCTGTTGGAATTACTGCTTTTTTATTACTATTATCATTACCCGTTTTAGCA:300
Sn-JF274201 GGGATTTCATCTATTCTAGGAGCTATTAATTTTATTACAACAATTATTAATATACGATTAAATAACTTATCATTTGATCAAATACCATTATTTATTTGAGCTGTTGGAATTACTGCTTTTTTATTACTATTATCATTACCCGTTTTAGCA:300
Sn-JF274203 GGGATTTCATCTATTCTAGGAGCTATTAATTTTATTACAACAATTATTAATATACGATTAAATAACTTATCATTTGATCAAATACCATTATTTATTTGAGCTGTTGGAATTACTGCTTTTTTATTACTATTATCATTACCCGTTTTAGCA:300
Sn-JF274199 GGGATTTCATCTATTCTAGGAGCTATTAATTTTATTACAACAATTATTAATATACGATTAAATAATTTATCATTTGATCAAATACCATTATTTATTTGAGCTGTTGGAATTACTGCTTTTTTATTACTATTATCATTACCCGTTTTAGCA:300
Sn-JF274205 GGAATTTCATCTATTCTAGGAGCTATTAATTTTATTACAACAATTATTAATATACGATTAAATAATTTATCATTTGATCAAATACCATTATTTATTTGAGCTGTTGGAATTACTGCTTTTTTATTACTATTATCATTACCCGTTTTAGCA:300
Sn-JF274204 GGAATTTCATCTATTCTAGGAGCTATTAATTTTATTACAACAATTATTAATATACGATTAAATAATTTATCATTTGATCAAATACCATTATTTATTTGAGCTGTTGGAATTACTGCTTTTTTATTACTATTATCATTACCCGTTTTAGCA:300
Sn-JF274192 GGGATTTCATCTATTCTAGGAGCTATTAATTTTATTACAACAATTATTAATATACGATTAAATAATTTATCATTTGATCAAATACCATTATTTATTTGAGCTGTTGGAATTACTGCTTTTTTATTACTACTATCATTACCCGTTTTAGCA:300
Sn-JF274181 GGAATTTCATCTATTCTAGGAGCTATTAATTTTATTACAACAATTATTAATATACGATTAAATAATTTATCATTTGATCAAATACCATTATTTATTTGAGCTGTTGGAATTACTGCTTTTTTATTACTATTATCATTACCCGTTTTAGCA:300
Sn-JF274198 GGGATTTCATCTATTCTAGGAGCTATTAATTTTATTACAACAATTATTAATATACGATTAAATAATTTATCATTTGATCAAATACCATTATTTATTTGAGCTGTTGGAATTACTGCTTTTTTATTACTATTATCATTACCCGTTTTAGCA:300
Sn-JF274195 GGGATTTCATCTATTCTAGGAGCTATTAATTTTATTACAACAATTATTAATATACGATTAAATAATTTATCATTTGATCAAATACCATTATTTATTTGAGCTGTTGGAATTACTGCTTTTTTATTACTATTATCATTACCCGTTTTAGCA:300
Sn-AJ829718 GGAATTTCATCTATTCTAGGAGCTATTAATTTTATTACAACAATTATTAATATATGATTAAATAATTTATCATTTGATCAAATACCATTATTTATTTGAGCTGTTGGAATTACTGCTTTTTTATTACTATTATCATTACCCGTTTTAGCA:300
Sn-AY649322 GGAATTTCATCTATTCTAGGAGCTATTAATTTTATTACAACAATTATTAATATACGATTAAATAATTTATCATTTGATCAAATACCATTATTTATTTGAGCTGTTGGAATTACTGCTTTTTTATTACTATTATCATTACCCGTTTTAGCA:300
Sn-JF274196 GGGATTTCATCTATTCTAGGAGCTATTAATTTTATTACAACAATTATTAATATACGATTAAATAACTTATCATTTGATCAAATACCATTATTTATTTGAGCTGTTGGAATTACTGCTTTTTTATTACTATTATCATTACCCGTTTTAGCA:300
Sn-JF274193 GGGATTTCATCTATTCTAGGAGCTATTAATTTTATTACAACAATTATTAATATACGATTAAATAATTTATCATTTGATCAAATACCATTATTTATTTGAGCTGTTGGAATTACTGCTTTTTTATTACTATTATCATTACCCGTTTTAGCA:300
Sn-JF274184 GGGATTTCATCTATTCTAGGAGCTATTAATTTTATTACAACAATTATTAATATACGATTAAATAATTTATCATTTGATCAAATACCATTATTTATTTGAGCTGTTGGAATTACTGCTTTTTTATTACTATTATCATTACCCGTTTTAGCA:300
Sn-JF274191 GGGATTTCATCTATTCTAGGAGCTATTAATTTTATTACAACAATTATTAATATACGATTAAATAATTTATCATTTGATCAAATACCATTATTTATTTGAGCTGTTGGAATTACTGCTTTTTTATTACTATTATCATTACCCGTTTTAGCA:300
Sn-JF274183 GGGATTTCATCTATTCTAGGAGCTATTAATTTTATTACAACAATTATTAATATACGATTAAATAATTTATCATTTGATCAAATACCATTATTTATTTGAGCTGTTGGAATTACTGCTTTTTTATTACTATTATCATTACCCGTTTTAGCA:300
Sn-JF274169 GGAATTTCATCTATTCTAGGAGCTATTAATTTTATTACAACAATTATTAATATACGATTAAATAATTTATCATTTGATCAAATACCATTATTTATTTGAGCTGTTGGAATTACTGCTTTTTTATTACTATTATCATTACCCGTTTTAGCA:300
Sn-F274188 GGGATTTCATCTATTCTAGGAGCTATTAATTTTATTACAACAATTATTAATATACGATTAAATAATTTATCATTTGATCAAATACCATTATTTATTTGAGCTGTTGGAATTACTGCTTTTTTATTACTATTATCATTACCCGTTTTAGCA:300
Sn-JF274186 GGGATTTCATCTATTCTAGGAGCTATTAATTTTATTACAACAATTATTAATATACGATTAAATAATTTATCATTTGATCAAATACCATTATTTATTTGAGCTGTTGGAATTACTGCTTTTTTATTACTATTATCATTACCCGTTTTAGCA:300
Sn-JF274176 GGAATTTCATCTATTCTAGGAGCTATTAATTTTATTACAACAATTATTAATATACGATTAAATAATTTATCATTTGATCAAATACCATTATTTATTTGAGCTGTTGGAATTACTGCTTTTTTATTACTATTATCATTACCCGTTTTAGCA:300
Sn-AJ829716 GGAATTTCATCTATTCTAGGAGCTATTAATTTTATTACAACAATTATTAATATACGATTAAATAATTTATCATTTGATCAAATACCATTATTTATTTGAGCTGTTGGAATTACTGCTTTTTTATTACTATTATCATTACCCGTTTTAGCA:300
Sn-JF274175 GGAATTTCATCTATTCTAGGAGCTATTAATTTTATTACAACAATTATTAATATACGATTAAATAATTTATCATTTGATCAAATACCATTATTTATTTGAGCTGTTGGAATTACTGCTTTTTTATTACTATTATCATTACCCGTTTTAGCA:300
Sn-JF274170 GGAATTTCATCTATTCTAGGAGCTATTAATTTTATTACAACAATTATTAATATACGATTAAATAATTTATCATTTGATCAAATACCATTATTTATTTGAGCTGTTGGAATTACTGCTTTTTTATTACTATTATCATTACCCGTTTTAGCA:300
Sn-AJ829715 GGAATTTCATCTATTCTAGGAGCTATTAATTTTATTACAACAATTATTAATATACGATTAAATAATTTATCATTTGATCAAATACCATTATTTATTTGAGCTGTTGGAATTACTGCTTTTTTATTACTATTATCATTACCCGTTTTAGCA:300
Sn-JF274182 GGGATTTCATCTATTCTAGGAGCTATTAATTTTATTACAACAATTATTAATATACGATTAAATAATTTATCATTTGATCAAATACCATTATTTATTTGAGCTGTTGGAATTACTGCTTTTTTATTACTATTATCATTACCCGTTTTAGCA:300
Sn-JF274194 GGGATTTCATCTATTCTAGGAGCTATTAATTTTATTACAACAATTATTAATATACGATTAAATAATTTATCATTTGATCAAATACCATTATTTATTTGAGCTGTTGGAATTACTGCTTTTTTATTACTATTATCATTACCCGTTTTAGCA:300
Sn-JF274202 GGGATTTCATCTATTCTAGGAGCTATTAATTTTATTACAACAATTATTAATATACGATTAAATAATTTATCATTTGATCAAATACCATTATTTATTTGAGCTGTTGGAATTACTGCTTTTTTATTACTATTATCATTACCCGTTTTAGCA:300
Sn-JF274187 GGGATTTCATCTATTCTAGGAGCTATTAATTTTATTACAACAATTATTAATATACGATTAAATAATTTATCATTTGATCAAATACCATTATTTATTTGAGCTGTTGGAATTACTGCTTTTTTATTACTATTATCATTACCCGTTTTAGCA:300
Sn-JF274172 GGAATTTCATCTATTCTAGGAGCTATTAATTTTATTACAACAATTATTAATATACGATTAAATAATTTATCATTTGATCAAATACCATTATTTATTTGAGCTGTTGGAATTACTGCTTTTTTATTACTATTATCATTACCCGTTTTAGCA:300
Sn-JF274168 GGAATTTCATCTATTCTAGGAGCTATTAATTTTATTACAACAATTATTAATATACGATTAAATAATTTATCATTTGATCAAATACCATTATTTATTTGAGCTGTTGGAATTACTGCTTTTTTATTACTATTATCATTACCCGTTTTAGCA:300
Sn-JF274197 GGGATTTCATCTATTCTAGGAGCTATTAATTTTATTACAACAATTATTAATATACGATTAAATAATTTATCATTTGATCAAATACCATTATTTATTTGAGCTGTTGGAATTACTGCTTTTTTATTACTATTATCATTACCCGTTTTAGCA:300
Sn-JF274185 GGGATTTCATCTATTCTAGGAGCTATTAATTTTATTACAACAATTATTAATATACGATTAAATAATTTATCATTTGATCAAATACCATTATTTATTTGAGCTGTTGGAATTACTGCTTTTTTATTACTATTATCATTACCCGTTTTAGCA:300
Sn-JF274200 GGGATTTCATCTATTCTAGGAGCTATTAATTTTATTACAACAATTATTAATATACGATTAAATAATTTATCATTTGATCAAATACCATTATTTATTTGAGCTGTTGGAATTACTGCTTTTTTATTACTATTATCATTACCCGTTTTAGCA:300
Sn-JF274180 GGGATTTCATCTATTCTAGGAGCTATTAATTTTATTACAACAATTATTAATATACGATTAAATAATTTATCATTTGATCAAATACCATTATTTATTTGAGCTGTTGGAATTACTGCTTTTTTATTACTATTATCATTACCCGTTTTAGCA:300
Sn-JF274171 GGAATTTCATCTATTCTAGGAGCTATTAATTTTATTACAACAATTATTAATATACGATTAAATAATTTATCATTTGATCAAATACCATTATTTATTTGAGCTGTTGGAATTACTGCTTTTTTATTACTATTATCATTACCCGTTTTAGCA:300
Sn-AJ829717 GGAATTTCATCTATTCTAGGAGCTATTAATTTTATTACAACAATTATTAATATACGATTAAATAATTTATCATTTGATCAAATACCATTATTTATTTGAGCTGTTGGAATTACTGCTTTTTTATTACTATTATCATTACCCGTTTTAGCA:300
Sn-JF274174 GGAATTTCATCTATTCTAGGAGCTATTAATTTTATTACAACAATTATTAATATACGATTAAATAATTTATCATTTGATCAAATACCATTATTTATTTGAGCTGTTGGAATTACTGCTTTTTTATTACTATTATCATTACCCGTTTTAGCA:300
Si-KP658205 GGTATTTCATCTATTTTAGGAGCTATTAATTTTATTACAACAATTATCAATATACGACTAAATAGTTTATCTTTTGATCAAATACCTCTATTTATTTGAGCTGTTGGAATTACTGCATTTTTATTATTATTATCTTTACCTGTATTAGCA:300
Si-MG838445 GGTATTTCATCTATTTTAGGAGCTATTAATTTTATTACAACAATTATCAATATACGACTAAATAGTTTATCTTTTGATCAAATACCTCTATTTATTTGAGCTGTTGGAATTACTGCATTTTTATTATTATTATCTTTACCTGTATTAGCA:300
Si-MT775880 GGTATTTCATCTATTTTAGGAGCTATTAATTTTATTACAACAATTATTAATATACGATTAAATAATTTATCCTTTGATCAAATACCTTTATTTATTTGAGCTGTTGGAATTACTGCATTTTTATTATTATTATCTTTACCTGTATTAGCA:300
Si-MT734563 GGTATTTCATCTATTTTAGGAGCTATTAATTTTATTACAACAATTATTAATATACGATTAAATAATTTATCCTTTGATCAAATACCTTTATTTATTTGAGCTGTTGGAATTACTGCATTTTTATTATTATTATCTTTACCTGTATTAGCA:300
Si-MT124087 GGTATTTCATCTATTTTAGGAGCTATTAATTTTATTACAACAATTATCAATATACGACTAAATAGTTTATCTTTTGATCAAATACCTCTATTTATTTGAGCTGTTGGAATTACTGCATTTTTATTATTATTATCTTTACCTGTATTAGCA:300
Si-MT124086 GGTATTTCATCTATTTTAGGAGCTATTAATTTTATTACAACAATTATCAATATACGACTAAATAGTTTATCTTTTGATCAAATACCTCTATTTATTTGAGCTGTTGGAATTACTGCATTTTTATTATTATTATCTTTACCTGTATTAGCA:300
Si-KT250763 GGTATTTCATCTATTTTAGGAGCTATTAATTTTATTACAACAATTATCAATATACGACTAAATAATTTATCTTTTGATCAAATACCTCTATTTATTTGAGCTGTTGGAATTACTGCATTTTTATTATTATTATCTTTACCTGTATTAGCA:300
Si-GU681997 GGTATTTCATCTATTTTAGGAGCTATTAATTTTATTACAACAATTATCAATATACGACTAAATAGTTTATCTTTTGATCAAATACCTCTATTTATTTGAGCTGTTGGAATTACTGCATTTTTATTATTATTATCTTTACCTGTATTAGCA:300
Si-MT734554 GGTATTTCATCTATTTTAGGAGCTATTAATTTTATTACTACAATTATTAATATACGATTAAATAGTTTATCTTTTGATCAAATACCTCTATTTATTTGAGCTGTTGGAATTACTGCATTTTTATTATTGCTATCTTTACCTGTTTTAGCA:300
Si-MT734546 GGTATTTCATCTATTTTAGGAGCTATTAATTTTATTACAACAATTATTAATATACGATTAAATAATTTATCCTTTGATCAAATACCTTTATTTATTTGAGCTGTTGGAATTACTGCATTTTTATTATTACTATCTTTACCTGTATTAGCA:300
Sca-MH851084 GGGATTTCATCTATTTTAGGAGCAATTAATTTTATTACAACAATTATTAATATACGATTAAATAGCCTATCATTTGATCAAATACCCCTATTCATTTGGGCTGTTGGAATTACTGCCTTTTTATTACTATTATCTTTACCTGTTTTAGCG:300
Sca-OR596544 GGAATTTCATCTATTTTAGGAGCAATTAATTTTATTACAACAATTATTAATATACGATTAAATAGCCTATCATTTGATCAAATACCCCTATTCATTTGGGCTGTTGGAATTACTGCCTTTTTATTACTATTATCTTTACCTGTTTTAGCG:300
Sca-OR596545 GGGATTTCATCTATTTTAGGAGCAATTAATTTTATTACAACAATTATTAATATACGATTAAATAGCCTATCATTTGATCAAATACCCCTATTCATTTGGGCTGTTGGAATTACTGCCTTTTTATTACTATTATCTTTACCTGTTTTAGCG:300
Sca-MH851102 GGAATTTCATCTATTTTAGGAGCAATTAATTTTATTACAACAATTATTAATATACGATTAAATAGCCTATCATTTGATCAAATACCCCTATTCATTTGGGCTGTTGGAATTACTGCCTTTTTATTACTATTATCTTTACCTGTTTTAGCG:300
Sca-KP682634 GGGATTTCATCTATTTTAGGAGCAATTAATTTTATTACAACAATTATTAATATACGATTAAATAGTCTATCATTCGATCAAATACCCCTATTCATTTGGGCTGTTGGAATTACTGCCTTTTTATTACTATTATCTTTACCTGTTTTAGCG:300
Sca-MK566706 GGGATTTCATCTATTTTAGGAGCAATTAATTTTATTACAACAATTATTAATATACGATTAAATAGTCTATCATTCGATCAAATACCCCTATTCATTTGGGCTGTTGGAATTACTGCCTTTTTATTACTATTATCTTTACCTGTTTTAGCG:300
Sca-MH851092 GGAATTTCATCTATTTTAGGAGCAATTAATTTTATTACAACAATTATTAATATACGATTAAATAGTCTATCATTCGATCAAATACCCCTATTCATTTGGGCTGTTGGAATTACTGCCTTTTTATTACTATTATCTTTACCTGTTTTAGCG:300
Sca-KF972231 GGGATTTCATCTATTTTAGGAGCAATTAATTTTATTACAACAATTATTAATATACGATTAAATAGTCTATCATTCGATCAAATACCCCTATTCATTTGGGCTGTTGGAATTACTGCCTTTTTATTACTATTATCTTTACCTGTTTTAGCG:300
Sca-MH851091 GGGATTTCATCTATTTTAGGGGCAATTAATTTTATTACAACAATTATTAATATACGATTAAATAGCCTATCATTTGATCAAATACCCCTATTCATTTGGGCTGTTGGAATTACTGCCTTTTTATTACTATTATCTTTACCTGTTTTAGCG:300
Sca-MH851099 GGGATTTCATCTATTTTAGGAGCAATTAATTTTATTACAACAATTATTAATATACGATTAAATAGTCTATCATTTGATCAAATACCCCTATTCATTTGGGCTGTTGGAATTACTGCCTTTTTATTACTATTATCTTTACCTGTTTTAGCG:300
Sa-MH851080 GGTATCTCTTCTATCTTAGGAGCTATTAATTTTATTACAACAATTATTAATATACGATTAAATAATTTATCATTTGATCAAATACCATTATTTATTTGAGCTGTGGGAATTACCGCATTTTTATTATTGTTATCATTACCAGTTTTAGCT:300
Sa-MH851079 GGTATCTCTTCTATCTTAGGAGCTATTAATTTTATTACAACAATTATTAATATACGATTAAATAATTTATCATTTGATCAAATACCATTATTTATTTGAGCTGTGGGAATTACCGCATTTTTATTATTGTTATCATTACCAGTTTTAGCT:300


 * 320 * 340 * 360 * 380 * 400 * 420 *
Sc-KU891975 GGAGCTATTACAATATTATTAACAGATCGAAATTTAAATACATCATTCTTTGATCCTGCAGGAGGAGGTGATCCAATTTTATATCAACATTTATTTTGATTTTTTGGACACCCAGAAGTTTATATTTTAATTTTACC:437
Sc-KU891973 GGAGCTATTACAATATTATTAACAGATCGAAATTTAAATACATCATTCTTTGATCCTGCAGGAGGAGGTGATCCAATTTTATATCAACATTTATTTTGATTTTTTGGACACCCAGAAGTTTATATTTTAATTTTACC:437
Sc-KU891972 GGAGCTATTACAATATTATTAACAGATCGAAATTTAAATACATCATTCTTTGATCCTGCAGGAGGAGGTGATCCAATTTTATATCAACATTTATTTTGATTTTTTGGACACCCAGAAGTTTATATTTTAATTTTACC:437
Sc-OM350099 GGAGCTATTACAATATTATTAACAGATCGAAATTTAAATACATCATTCTTTGATCCTGCAGGAGGAGGTGATCCAATTTTATATCAACATTTATTTTGATTTTTTGGACACCCAGAAGTTTATATTTTAATTTTACC:437
Sc-OM350097 GGAGCTATTACAATATTATTAACAGATCGAAATTTAAATACATCATTCTTTGATCCTGCAGGAGGAGGTGATCCAATTTTATATCAACATTTATTTTGATTTTTTGGACACCCAGAAGTTTATATTTTAATTTTACC:437
Sc-OM350096 GGAGCTATTACAATATTATTAACAGATCGAAATTTAAATACATCATTCTTTGATCCTGCAGGAGGAGGTGATCCAATTTTATATCAACATTTATTTTGATTTTTTGGACACCCAGAAGTTTATATTTTAATTTTACC:437
Sc-MH851121 GGAGCTATTACAATATTATTAACAGATCGAAATTTAAATACATCATTCTTTGATCCTGCAGGAGGAGGTGATCCAATTTTATATCAACATTTATTTTGATTTTTTGGACACCCAGAAGTTTATATTTTAATTTTACC:437
Sc-PV646697 GGAGCTATTACAATATTATTAACAGATCGAAATTTAAATACATCATTCTTTGATCCTGCAGGAGGAGGTGATCCAATTTTATATCAACATTTATTTTGATTTTTTGGACACCCAGAAGTTTATATTTTAATTTTACC:437
Sc-KU891978 GGAGCTATTACAATATTATTAACAGATCGAAATTTAAATACATCATTCTTTGATCCTGCAGGAGGAGGTGATCCAATTTTATATCAACATTTATTTTGATTTTTTGGACACCCAGAAGTTTATATTTTAATTTTACC:437
Sc-KU891976 GGAGCTATTACAATATTATTAACAGATCGAAATTTAAATACATCATTCTTTGATCCTGCAGGAGGAGGTGATCCAATTTTATATCAACATTTATTTTGATTTTTTGGACACCCAGAAGTTTATATTTTAATTTTACC:437
Sc-KU891977 GGAGCTATTACAATATTATTAACAGATCGAAATTTAAATACATCATTCTTTGATCCTGCAGGAGGAGGTGATCCAATTTTATATCAACATTTATTTTGATTTTTTGGACACCCAGAAGTTTATATTTTAATTTTACC:437
Sc-OR758844 GGAGCTATTACAATATTATTAACAGATCGAAATTTAAATACATCATTCTTTGATCCTGCAGGAGGAGGTGATCCAATTTTATATCAACATTTATTTTGATTTTTTGGACACCCAGAAGTTTATATTTTAATTTTACC:437
Sc-GWOSA261-10 GGAGCTATTACAATATTATTAACAGATCGAAATTTAAATACATCATTCTTTGATCCTGCAGGAGGAGGTGATCCAATTTTATATCAACATTTATTTTGATTTTTTGGACACCCAGAAGTTTATATTTTAATTTTACC:437
Sc-GWOSA262-10 GGAGCTATTACAATATTATTAACAGATCGAAATTTAAATACATCATTCTTTGATCCTGCAGGAGGAGGTGATCCAATTTTATACCAACATTTATTTTGATTTTTTGGACACCCAGAAGTTTATATTTTAATTTTACC:437
Sc-GBMIN83467-17 GGAGCTATTACAATATTATTAACAGATCGAAATTTAAATACATCATTCTTTGATCCTGCAGGAGGAGGTGATCCAATTTTATATCAACATTTATTTTGATTTTTTGGACACCCAGAAGTTTATATTTTAATTTTACC:437
Sc-GBMIN83468-17 GGAGCTATTACAATATTATTAACAGATCGAAATTTAAATACATCATTCTTTGATCCTGCAGGAGGAGGTGATCCAATTTTATATCAACATTTATTTTGATTTTTTGGACACCCAGAAGTTTATATTTTAATTTTACC:437
Sc-GBGL20669-18 GGAGCTATTACAATATTATTAACAGATCGAAATTTAAATACATCATTCTTTGATCCTGCAGGAGGAGGTGATCCAATTTTATATCAACATTTATTTTGATTTTTTGGACACCCAGAAGTTTATATTTTAATTTTACC:437
Sc-GBGL20668-18 GGAGCTATTACAATATTATTAACAGATCGAAATTTAAATACATCATTCTTTGATCCTGCAGGAGGAGGTGATCCAATTTTATATCAACATTTATTTTGATTTTTTGGACACCCAGAAGTTTATATTTTAATTTTACC:437
Sc-LNAUY1377-19 GGAGCTATTACAATATTATTAACAGATCGAAATTTAAATACATCATTCTTTGATCCTGCAGGAGGAGGTGATCCAATTTTATATCAACATTTATTTTGATTTTTTGGACACCCAGAAGTTTATATTTTAATTTTACC:437
Sc-GBGL24680-19 GGAGCTATTACAATATTATTAACAGATCGAAATTTAAATACATCATTCTTTGATCCTGCAGGAGGAGGTGATCCAATTTTATATCAACATTTATTTTGATTTTTTGGACACCCAGAAGTTTATATTTTAATTTTACC:437
Sc-GBGL24681-19 GGAGCTATTACAATATTATTAACAGATCGAAATTTAAATACATCATTCTTTGATCCTGCAGGAGGAGGTGATCCAATTTTATATCAACATTTATTTTGATTTTTTGGACACCCAGAAGTTTATATTTTAATTTTACC:437
Sc-LNAUY1379-19 GGAGCTATTACAATATTATTAACAGATCGAAATTTAAATACATCATTCTTTGATCCTGCAGGAGGAGGTGATCCAATTTTATATCAACATTTATTTTGATTTTTTGGACACCCAGAAGTTTATATTTTAATTTTACC:437
Sc-GBGL24682-19 GGAGCTATTACAATATTATTAACAGATCGAAATTTAAATACATCATTCTTTGATCCTGCAGGAGGAGGTGATCCAATTTTATATCAACATTTATTTTGATTTTTTGGACACCCAGAAGTTTATATTTTAATTTTACC:437
Sc-LNAUY1378-19 GGAGCTATTACAATATTATTAACAGATCGAAATTTAAATACATCATTCTTTGATCCTGCAGGAGGAGGTGATCCAATTTTATATCAACATTTATTTTGATTTTTTGGACACCCAGAAGTTTATATTTTAATTTTACC:437
Sc-GBGL24687-19 GGAGCTATTACAATATTATTAACAGATCGAAATTTAAATACATCATTCTTTGATCCTGCAGGAGGAGGTGATCCAATTTTATATCAACATTTATTTTGATTTTTTGGACACCCAGAAGTTTATATTTTAATTTTACC:437
Sc-GBGL24686-19 GGAGCTATTACAATATTATTAACAGATCGAAATTTAAATACATCATTCTTTGATCCTGCAGGAGGAGGTGATCCAATTTTATATCAACATTTATTTTGATTTTTTGGACACCCAGAAGTTTATATTTTAATTTTACC:437
Sc-GBMNE61282-22 GGAGCTATTACAATATTATTAACAGATCGAAATTTAAATACATCATTCTTTGATCCTGCAGGAGGAGGTGATCCAATTTTATATCAACATTTATTTTGATTTTTTGGACACCCAGAAGTTTATATTTTAATTTTACC:437
Sc-GBMNE61283-22 GGAGCTATTACAATATTATTAACAGATCGAAATTTAAATACATCATTCTTTGATCCTGCAGGAGGAGGTGATCCAATTTTATATCAACATTTATTTTGATTTTTTGGACACCCAGAAGTTTATATTTTAATTTTACC:437
Sc-GBMNE61285-22 GGAGCTATTACAATATTATTAACAGATCGAAATTTAAATACATCATTCTTTGATCCTGCAGGAGGAGGTGATCCAATTTTATATCAACATTTATTTTGATTTTTTGGACACCCAGAAGTTTATATTTTAATTTTACC:437
Sc-GBAAW8767-24 GGAGCTATTACAATATTATTAACAGATCGAAATTTAAATACATCATTCTTTGATCCTGCAGGAGGAGGTGATCCAATTTTATATCAACATTTATTTTGATTTTTTGGACACCCAGAAGTTTATATTTTAATTTTACC:437
Sn-GBGL12313-13 GGAGCTATTACTATACTACTTACAGATCGAAATTTAAACACATCATTTTTTGATCCTGCGGGAGGAGGTGATCCAATTTTATACCAACACTTATTCTGATTTTTTGGGCATCCAGAAGTTTATATTTTAATTTTACC:437
Sn-JF274189 GGAGCTATTACTATATTACTTACAGATCGAAATTTAAATACATCATTTTTTGATCCTGCGGGAGGAGGTGATCCAATTTTATACCAACACTTATTCTGATTTTTTGGGCATCCAGAAGTTTATATTTTAATTTTACC:437
Sn-JF274179 GGAGCTATTACTATACTACTTACGGATCGAAATTTAAATACATCATTTTTTGATCCTGCGGGAGGAGGTGATCCAATTTTATACCAACACTTATTCTGATTTTTTGGGCATCCAGAAGTTTATATTTTAATTTTACC:437
Sn-JF274178 GGAGCTATTACTATACTACTTACGGATCGAAATTTAAATACATCATTTTTTGATCCTGCGGGAGGAGGTGATCCAATTTTATACCAACACTTATTCTGATTTTTTGGGCATCCAGAAGTTTATATTTTAATTTTACC:437
Sn-JF274177 GGAGCTATTACTATACTACTTACGGATCGAAATTTAAATACATCATTTTTTGATCCTGCGGGAGGAGGTGATCCAATTTTATACCAACACTTATTCTGATTTTTTGGGCATCCAGAAGTTTATATTTTAATTTTACC:437
Sn-JF274173 GGAGCTATTACTATACTACTTACGGATCGAAATTTAAATACATCATTTTTTGATCCTGCGGGAGGAGGTGATCCAATTTTATACCAACACTTATTCTGATTTTTTGGACATCCAGAAGTTTATATTTTAATTTTACC:437
Sn-JF274201 GGAGCTATTACTATACTACTTACGGATCGAAATTTAAATACATCGTTTTTTGATCCTGCAGGAGGAGGTGATCCAATTTTATACCAACACTTATTCTGATTTTTTGGGCATCCAGAAGTTTATATTTTAATTTTACC:437
Sn-JF274203 GGAGCTATTACTATACTACTTACGGATCGAAATTTAAATACATCATTTTTTGATCCTGCAGGAGGAGGTGATCCAATTTTATACCAACACTTATTCTGATTTTTTGGGCATCCAGAAGTTTATATTTTAATTTTACC:437
Sn-JF274199 GGAGCTATTACTATACTACTTACGGATCGAAATTTAAATACATCATTTTTTGATCCTGCAGGAGGAGGTGATCCAATTTTATACCAACACTTATTCTGATTTTTTGGGCATCCAGAAGTTTATATTTTAATTTTACC:437
Sn-JF274205 GGAGCTATTACTATACTACTTACGGATCGAAATTTAAATACATCATTTTTTGATCCTGCAGGAGGGGGTGATCCAATTTTATACCAACACCTATTCTGATTTTTTGGACACCCAGAAGTTTATATTTTAATTTTACC:437
Sn-JF274204 GGAGCTATTACTATACTACTTACGGATCGAAATTTAAATACATCATTTTTTGATCCTGCAGGAGGGGGTGATCCAATTTTATACCAACACCTATTCTGATTTTTTGGGCACCCAGAAGTTTATATTTTAATTTTACC:437
Sn-JF274192 GGAGCTATTACTATACTACTTACGGATCGAAATTTAAATACATCATTTTTTGATCCTGCAGGAGGAGGTGATCCAATTTTATACCAACACTTATTCTGATTTTTTGGGCATCCAGAAGTTTATATTTTAATTTTACC:437
Sn-JF274181 GGAGCTATTACTATACTACTTACGGATCGAAATTTAAATACATCATTTTTTGATCCTGCGGGAGGAGGTGATCCAATTTTATACCAACACTTATTCTGATTTTTTGGGCATCCAGAAGTTTATATTTTAATTTTACC:437
Sn-JF274198 GGAGCTATTACTATACTACTTACGGATCGAAATTTAAATACATCATTTTTTGATCCTGCAGGAGGAGGTGATCCAATTTTATACCAACACTTATTCTGATTTTTTGGGCATCCAGAAGTTTATATTTTAATTTTACC:437
Sn-JF274195 GGAGCTATTACTATACTACTTACGGATCGAAATTTAAATACATCATTTTTTGATCCTGCAGGAGGAGGTGATCCAATTTTATACCAACACTTATTCTGATTTTTTGGACATCCAGAAGTTTATATTTTAATTTTACC:437
Sn-AJ829718 GGAGCTATTACTATACTACTTACGGATCGAAATTTAAATACATCATTTTTTGATCCTGCGGGAGGAGGTGATCCAATTTTATACCAACACTTATTCTGATTTTTTGGACATCCAGAAGTTTATATTTTAATTTTACC:437
Sn-AY649322 GGAGCTATTACTATACTACTTACGGATCGAAATTTAAATACATCATTTTTTGATCCTGCGGGAGGAGGTGATCCAATTTTATACCAACACTTATTCTGATTTTTTGGACATCCAGAAGTTTATATTTTAATTTTACC:437
Sn-JF274196 GGAGCTATTACTATACTACTTACGGATCGAAATTTAAATACATCATTTTTTGATCCTGCAGGAGGAGGTGATCCAATTTTATACCAACACTTATTCTGATTTTTTGGGCATCCAGAAGTTTATATTTTAATTTTACC:437
Sn-JF274193 GGAGCTATTACTATACTACTTACGGATCGAAATTTAAATACATCATTTTTTGATCCTGCAGGAGGAGGTGATCCAATTTTATATCAACACTTATTCTGATTTTTTGGGCATCCAGAAGTTTATATTTTAATTTTACC:437
Sn-JF274184 GGAGCTATTACTATACTACTTACAGATCGAAATTTAAATACATCATTTTTTGATCCTGCGGGAGGAGGTGATCCAATTTTATACCAACACTTATTCTGATTTTTTGGACATCCAGAAGTTTATATTTTAATTTTACC:437
Sn-JF274191 GGAGCTATTACTATACTACTTACAGATCGAAATTTAAATACATCATTTTTTGATCCTGCGGGAGGAGGTGATCCAATTTTATACCAACACTTATTCTGATTTTTTGGGCATCCAGAAGTTTATATTTTAATTTTACC:437
Sn-JF274183 GGAGCTATTACTATACTACTTACAGATCGAAATTTAAATACATCATTTTTTGATCCTGCGGGAGGAGGTGATCCAATTTTATACCAACACTTATTCTGATTTTTTGGGCATCCAGAAGTTTATATTTTAATTTTACC:437
Sn-JF274169 GGAGCTATTACTATACTACTTACGGATCGAAATTTAAATACATCATTTTTTGATCCTGCGGGAGGAGGTGATCCAATTTTATACCAACACTTATTCTGATTTTTTGGACATCCAGAAGTTTATATTTTAATTTTACC:437
Sn-F274188 GGAGCTATTACTATACTACTTACGGATCGAAATTTAAATACATCATTTTTTGATCCTGCGGGGGGAGGTGATCCAATTTTATACCAACACTTATTCTGATTTTTTGGGCATCCAGAAGTTTATATTTTAATTTTACC:437
Sn-JF274186 GGAGCTATTACTATACTACTTACGGATCGAAATTTAAATACATCATTTTTTGATCCTGCGGGAGGAGGTGATCCAATTTTATACCAACACTTATTCTGATTTTTTGGGCATCCAGAAGTTTATATTTTAATTTTACC:437
Sn-JF274176 GGAGCTATTACTATACTACTTACGGATCGAAATTTAAATACATCATTTTTTGATCCTGCGGGAGGAGGTGATCCAATTTTATACCAACACTTATTCTGATTTTTTGGACATCCAGAAGTTTATATTTTAATTTTACC:437
Sn-AJ829716 GGAGCTATTACTATACTACTTACGGATCGAAATTTAAATACATCATTTTTTGATCCTGCGGGAGGAGGTGATCCAATTTTATACCAACACTTATTCTGATTTTTTGGACATCCAGAAGTTTATATTTTAATTTTACC:437
Sn-JF274175 GGAGCTATTACTATACTACTTACGGATCGAAATTTAAATACATCATTTTTTGATCCTGCGGGAGGAGGTGATCCAATTTTATACCAACACTTATTCTGATTTTTTGGACATCCAGAAGTTTATATTTTAATTTTACC:437
Sn-JF274170 GGAGCTATTACTATACTACTTACGGATCGAAATTTAAATACATCATTTTTTGATCCTGCGGGAGGAGGTGATCCAATTTTATACCAACACTTATTCTGATTTTTTGGACATCCAGAAGTTTATATTTTAATTTTACC:437
Sn-AJ829715 GGAGCTATTACTATACTACTTACGGATCGAAATTTAAATACATCATTTTTTGATCCTGCGGGAGGAGGTGATCCAATTTTATACCAACACTTATTCTGATTTTTTGGACATCCAGAAGTTTATATTTTAATTTTACC:437
Sn-JF274182 GGAGCTATTACTATACTACTTACGGATCGAAATTTAAATACATCATTTTTTGATCCTGCGGGAGGAGGTGATCCAATTTTATACCAACACTTATTCTGATTTTTTGGGCATCCAGAAGTTTATATTTTAATTTTACC:437
Sn-JF274194 GGAGCTATTACTATACTACTTACGGATCGAAATTTAAATACATCATTTTTTGATCCTGCAGGAGGAGGTGATCCAATTTTATACCAACACTTATTCTGATTTTTTGGGCATCCAGAAGTTTATATTTTAATTTTACC:437
Sn-JF274202 GGAGCTATTACTATACTACTTACGGATCGAAATTTAAATACATCATTTTTTGATCCTGCGGGAGGAGGTGATCCAATTTTATACCAACACTTATTCTGATTTTTTGGGCATCCAGAAGTTTATATTTTAATTTTACC:437
Sn-JF274187 GGAGCTATTACTATACTACTTACGGATCGAAATTTAAATACATCATTTTTTGATCCTGCGGGAGGAGGTGATCCAATTTTATACCAACACTTATTCTGATTTTTTGGACATCCAGAAGTTTATATTTTAATTTTACC:437
Sn-JF274172 GGAGCTATTACTATACTACTTACAGATCGAAATTTAAATACATCATTTTTTGATCCTGCGGGAGGAGGTGATCCAATTTTATACCAACACTTATTCTGATTTTTTGGACATCCAGAAGTTTATATTTTAATTTTACC:437
Sn-JF274168 GGAGCTATTACTATACTACTTACGGATCGAAATTTAAATACATCATTTTTTGATCCTGCGGGAGGAGGTGATCCAATTTTATACCAACACTTATTCTGATTTTTTGGACATCCAGAAGTTTATATTTTAATTTTACC:437
Sn-JF274197 GGAGCTATTACTATACTACTTACGGATCGAAATTTAAATACATCATTTTTTGATCCTGCAGGAGGAGGTGATCCAATTTTATACCAACACTTATTCTGATTTTTTGGACATCCAGAAGTTTATATTTTAATTTTACC:437
Sn-JF274185 GGAGCTATTACTATACTACTTACGGATCGAAATTTAAATACATCATTTTTTGATCCTGCGGGAGGAGGTGACCCAATTTTATACCAACACTTATTCTGATTTTTTGGGCATCCAGAAGTTTATATTTTAATTTTACC:437
Sn-JF274200 GGAGCTATTACTATACTACTTACGGATCGAAATTTAAATACATCATTTTTTGATCCTGCAGGAGGAGGTGATCCAATTTTATACCAACACTTATTCTGATTTTTTGGACATCCAGAAGTTTATATTTTAATTTTACC:437
Sn-JF274180 GGAGCTATTACTATACTACTTACGGATCGAAATTTAAATACATCATTTTTTGATCCTGCGGGAGGAGGTGATCCAATTTTATACCAACACTTATTCTGATTTTTTGGGCATCCAGAAGTTTATATTTTAATTTTACC:437
Sn-JF274171 GGAGCTATTACTATACTACTTACGGATCGAAATTTAAATACATCATTTTTTGATCCTGCGGGAGGAGGTGATCCAATTTTATACCAACACTTATTCTGATTTTTTGGACATCCAGAAGTTTATATTTTAATTTTACC:437
Sn-AJ829717 GGAGCTATTACTATACTACTTACGGATCGAAATTTAAATACATCATTTTTTGATCCTGCGGGAGGAGGTGATCCAATTTTATACCAACACTTATTCTGATTTTTTGGACATCCAGAAGTTTATATTTTAATTTTACC:437
Sn-JF274174 GGAGCTATTACTATACTACTTACGGATCGAAATTTAAATACATCATTTTTTGATCCTGCGGGAGGAGGTGATCCAATTTTATACCAACACTTATTCTGATTTTTTGGACATCCAGAAGTTTATATTTTAATTTTACC:437
Si-KP658205 GGAGCTATTACAATATTATTAACAGATCGAAATTTAAATACATCATTCTTTGACCCTGCAGGAGGAGGTGATCCTATTTTATACCAACATTTATTT-----------------------------------------:396
Si-MG838445 GGAGCTATTACAATATTATTAACAGATCGAAATTTAAATACATCATTCTTTGATCCTGCAGGAGGAGGTGATCCTATTTTATACCAACATTTATTT-----------------------------------------:396
Si-MT775880 GGAGCTATTACAATATTGTTAACAGATCGAAATTTAAACACATCATTCTTTGATCCTGCAGGAGGAGGGGACCCTATTTTATATCAACACTTATTT-----------------------------------------:396
Si-MT734563 GGAGCTATTACAATATTGTTAACAGATCGAAATTTAAACACATCATTCTTTGATCCTGCAGGAGGAGGGGACCCTATTTTATATCAACACTTATTT-----------------------------------------:396
Si-MT124087 GGAGCTATTACAATATTATTGACAGATCGAAATTTAAATACATCATTCTTTGACCCTGCAGGAGGAGGTGATCCTATTTTATACCAACATTTATTT-----------------------------------------:396
Si-MT124086 GGAGCTATTACAATATTATTGACAGATCGAAATTTAAATACATCATTCTTTGACCCTGCAGGAGGAGGTGATCCTATTTTATACCAACATTTATTT-----------------------------------------:396
Si-KT250763 GGAGCTATTACAATATTATTGACAGATCGAAATTTAAATACATCATTCTTTGACCCCGCAGGAGGAGGTGATCCTATTTTATACCAACATTTATTT-----------------------------------------:396
Si-GU681997 GGAGCTATTACAATATTATTAACAGATCGAAATTTAAATACATCATTCTTTGACCCCGCAGGAGGAGGTGATCCTATTTTATACCAACATTTATTT-----------------------------------------:396
Si-MT734554 GGAGCTATTACAATATTATTAACAGATCGAAATTTAAATACATCATTCTTTGACCCTGCAGGAGGGGGTGACCCTATTTTATATCAACATTTATTT-----------------------------------------:396
Si-MT734546 GGAGCTATTACAATATTACTAACAGATCGAAATTTAAATACATCATTCTTTGATCCTGCAGGAGGAGGGGACCCTATTTTATATCAACATTTATTT-----------------------------------------:396
Sca-MH851084 GGAGCCATTACTATATTACTTACAGATCGAAATTTAAATACTTCATTTTTTGATCCTGCAGGAGGAGGAGATCCAATTTTATATCAACACTTATTT-----------------------------------------:396
Sca-OR596544 GGAGCCATTACTATATTACTTACAGATCGAAATTTAAATACTTCATTTTTTGATCCTGCAGGAGGAGGAGATCCAATTTTATATCAACACTTATTT-----------------------------------------:396
Sca-OR596545 GGAGCCATTACTATATTACTTACAGATCGAAATTTAAATACTTCATTTTTTGATCCTGCAGGAGGAGGAGATCCAATTTTATATCAACACTTATTT-----------------------------------------:396
Sca-MH851102 GGAGCCATTACTATATTACTTACAGATCGAAATTTAAATACTTCATTTTTTGATCCTGCAGGAGGAGGAGATCCAATTTTATATCAACACTTATTT-----------------------------------------:396
Sca-KP682634 GGAGCTATTACTATATTACTTACAGATCGAAATTTAAATACTTCATTTTTTGATCCTGCAGGAGGAGGAGATCCAATTTTATATCAACACTTATTT-----------------------------------------:396
Sca-MK566706 GGAGCTATTACTATATTACTTACAGATCGAAATTTAAATACTTCATTTTTTGATCCTGCAGGAGGAGGAGATCCAATTTTATATCAACACTTATTT-----------------------------------------:396
Sca-MH851092 GGAGCTATTACTATATTACTTACAGATCGAAATTTAAATACTTCATTTTTTGATCCTGCAGGAGGAGGAGATCCAATTTTATATCAACACTTATTT-----------------------------------------:396
Sca-KF972231 GGAGCTATTACTATATTACTTACAGATCGAAATTTAAATACTTCATTTTTTGATCCTGCAGGAGGAGGAGATCCAATTTTATATCAACACTTATTT-----------------------------------------:396
Sca-MH851091 GGAGCCATTACTATATTACTTACAGATCGAAATTTAAATACTTCATTTTTTGATCCTGCAGGAGGAGGAGATCCAATTTTATATCAACACTTATTT-----------------------------------------:396
Sca-MH851099 GGAGCCATTACTATATTACTTACAGATCGAAATTTAAATACTTCATTTTTTGATCCTGCAGGAGGAGGAGATCCAATTTTATATCAACACTTATTT-----------------------------------------:396
Sa-MH851080 GGAGCTATTACAATATTATTAACAGACCGAAATTTAAATACATCATTTTTTGATCCAGCAGGAGGAGGAGATCCAATTTTATATCAACATTTATTC-----------------------------------------:396
Sa-MH851079 GGAGCTATTACAATATTATTAACAGACCGAAATTTAAATACATCATTTTTTGATCCAGCAGGAGGAGGAGATCCAATTTTATATCAACATTTATTC-----------------------------------------:396

**Supplementary Figure S1.** GeneDoc view of 437 bp partial *COI* gene alignment of *Sesamia cretica*, *S. nonagrioides*, and other congeneric *Sesamia* species retrieved from GenBank (abbriavted species name – GB accession number). Conserved and variable nucleotide positions are shown. Primer binding regions (*S. cretica* forward primer: yellow, *S. nonagrioides* forward primer: green, shared reverse primer for both species: blue) used for the species-specific multiplex PCR assay are highlighted. Dashes (—) indicate regions where homologous nucleotide positions were unavailable for certain *Sesamia* species in GenBank, resulting in truncated alignment at the 3′ end. The reverse primer was designed within this region, ensuring amplification specificity to *S. cretica* and *S. nonagrioides*. Species abbreviations correspond to: *S. cretica* (Sc), *S. nonagrioides* (Sn), *S. inferens* (Si), *S. calamistis* (Sca), and *S. albicolor* (Sa). Accession numbers follow GenBank records; some codes represent BOLD system Process IDs corresponding to barcode database entries.
